# Supplementary material for: Feasibility randomised controlled trial of online group Acceptance and Commitment Therapy for Functional Cognitive Disorder (ACT4FCD)
Source: BMJ Open. 2023 May 11;13(5):e072366. doi: 10.1136/bmjopen-2023-072366 (PMC10186420; doi:10.1136/bmjopen-2023-072366)
Supplement: Author's manuscript [file bmjopen-2023-072366.draft_revisions.pdf]

BMJ Open

BMJ Open is committed to open peer review. As part of this commitment we make the peer review history of every article we publish publicly available.

When an article is published we post the peer reviewers' comments and the authors' responses online. We also post the versions of the paper that were used during peer review. These are the versions that the peer review comments apply to.

The versions of the paper that follow are the versions that were submitted during the peer review process. They are not the versions of record or the final published versions. They should not be cited or distributed as the published version of this manuscript.

BMJ Open is an open access journal and the full, final, typeset and author-corrected version of record of the manuscript is available on our site with no access controls, subscription charges or pay-per-view fees (http://bmjopen.bmj.com).

If you have any questions on BMJ Open's open peer review process please email info.bmjopen@bmj.com

BMJ Open

Feasibility randomised controlled trial of online group Acceptance and Commitment Therapy for Functional Cognitive Disorder (ACT4FCD)

Journal:	*BMJ Open*
Manuscript ID	bmjopen-2023-072366
Article Type:	Protocol
Date Submitted by the Author:	20-Feb-2023
Complete List of Authors:	Poole, Norman; South West London and Saint George's Mental Health NHS Trust,<br>Cope, Sarah; South West London and Saint George's Mental Health NHS Trust<br>Vanzan, Serena; South West London and Saint George's Mental Health NHS Trust<br>Duffus, Aimee; South West London and Saint George's Mental Health NHS Trust<br>Mantovani, Nadia; St George's Hospital<br>Smith, Jared; St George's Hospital<br>Barrett, Barbara; King's College London<br>Tokley, Melanie; Eastern Community Mental Health Centre<br>Scicluna, Martin; South West London and Saint George's Mental Health NHS Trust<br>Beardmore, Sarah; South West London and Saint George's Mental Health NHS Trust<br>Turner, Kati; St George's University of London<br>Edwards, Mark; King's College London Institute of Psychiatry Psychology and Neuroscience<br>Howard, Robert; University College London Division of Psychiatry
Keywords:	PSYCHIATRY, NEUROLOGY, Delirium & cognitive disorders < PSYCHIATRY

SCHOLARONE™
Manuscripts

Feasibility randomised controlled trial of online group Acceptance and Commitment Therapy for Functional Cognitive Disorder (ACT4FCD)

For peer review only

Authors

Norman Poole,* Sarah Cope, Serena Vanzan, Aimee Duffus, Nadia Mantovani, Jared Smith, Barbara Barrett, Melanie Tokley, Martin Scicluna, Sarah Beardmore, Kati Turner, Mark Edwards, Rob Howard

Affiliations

NP: norman.poole@gmail.com (*corresponding author) South West London & St George's Mental Health NHS Trust; St George's Hospital Medical School (London, UK)

SC: sarah.cope@swlstg.nhs.uk South West London & St George's Mental Health NHS Trust (London, UK)

SV: serena.vanzan@swlstg.nhs.uk South West London & St George's Mental Health NHS Trust (London, UK)

AD: aimee.duffus@swlstg.nhs.uk South West London & St George's Mental Health NHS Trust (London, UK)

NM: nmantova@sgul.ac.uk South West London & St George's Mental Health NHS Trust; St George's Hospital Medical School (London, UK)

JS: jasmith@sgul.ac.uk South West London & St George's Mental Health NHS Trust; St George's Hospital Medical School (London, UK)

BB: barbara.m.barrett@kcl.ac.uk King's Health Economics, King's College London (London, UK)

MT: melanie.tokley@sa.gov.au Eastern Community Mental Health Centre (Adelaide, Australia); Rose Park Psychology (Adelaide, Australia)

MS: scicluna.mg@gmail.com PPI Rep, South West London & St George's Mental Health NHS Trust (London, UK)

SB: s_beardmore@hotmail.co.uk PPI Rep, South West London & St George's Mental Health NHS Trust (London, UK)

KT: creusk@aol.com South West London & St George's Mental Health NHS Trust; St George's Hospital Medical School (London, UK)

ME: mark.j.edwards@kcl.ac.uk Institute of Psychiatry, Psychology and Neuroscience, King's College London; South London and the Maudsley Hospital (London, UK)

RH: robert.howard@ucl.ac.uk University College London Division of Psychiatry (London, UK)

Word count

4021

Protocol version number & date

Version 2.0, 14/11/2022

For peer review only

Article Summary

Abstract

Introduction

Functional cognitive disorder is seen increasingly in clinics commissioned to assess cognitive disorders. Patients complain of frequent cognitive, especially memory, failures. The diagnosis can be made clinically, and unnecessary investigations avoided. While there is some evidence that psychological treatments can be helpful, they are not routinely available. Therefore, we have developed a brief psychological intervention using the principles of Acceptance and Commitment Therapy (ACT) that can be delivered in groups and online. We are conducting a feasibility study to assess whether the intervention can be delivered within a randomised controlled trial. We aim to study the feasibility of recruitment, willingness to be randomised to intervention or control condition, adherence to the intervention, completion of outcome measures, and acceptability of treatment.

Methods and analysis

We aim to recruit 48 participants randomised 50:50 to either the ACT intervention and Treatment as Usual (TAU), or TAU alone. ACT will be provided to participants in the treatment arm following completion of baseline outcome measures. Completion of these outcome measures will be repeated at 8, 16, and 26 weeks. The measures will assess several domains including psychological flexibility, subjective cognitive symptoms, mood and anxiety, health related quality of life and functioning, healthcare utilisation, and satisfaction with care and participant-rated improvement. Fifteen participants will be selected for in-depth qualitative interviews about their experiences of living with FCD and of the ACT intervention.

Ethics and dissemination

The study received a favourable opinion from the South East Scotland Research Ethics Committee 02 on 30th September 2022 (REC reference: 22/SS/0059). HRA approval was received on 1st November 2022 (IRAS 313730). The study has been registered with the ISRCTN (ISRCTN12939037). The results will be published in full in an open access journal.

For peer review only

ISRCTN Registration

https://doi.org/10.1186/ISRCTN12939037

Grant Number

National Institute for Health and Care Research (NIHR) grant number: NIHR202743

Strengths and limitations of this study

- Novel intervention with potential to be delivered at scale
- Adherence to principles of an evidence-based intervention (ACT)
- Utilising qualitative and quantitative methodologies which is key to contextualising patient experiences in a clinically meaningful measurement framework
- Recruiting from the full range of services that assess cognition in adults in the UK
- Participants not blinded to their intervention

Keywords

Functional Neurological Disorder

Cognitive Symptoms

Acceptance and Commitment Therapy

Randomized Controlled Trial

For peer review only

Introduction

Functional Cognitive Disorder (FCD) is defined as a complaint about memory function or other cognitive process in the absence of relevant neuropathology and with evidence of inconsistency between symptoms reported, objective signs, and known phenomenology of dementia syndromes.[1] Typical complaints include forgetting an intended action while in the process of carrying it out; inability to recall well-founded memories (such as PIN numbers and names); disruptions in the flow of thoughts and conversations; word-finding difficulties; and spoonerisms. [2] While anxiety and depression are common co-morbidities, they are generally mild and do not account for the severity or nature of the symptoms reported. Patients who experience FCD tend to perform as well or a little worse on neuropsychological tests as healthy controls but better than those with mild cognitive impairment and early Alzheimer's disease.[3] Disturbance of attention is thought to be responsible for the symptoms,[4] as with other functional neurological disorders,[5] although the underlying pathophysiological mechanisms remain unknown.

Diagnostic Memory Clinics (DMCs) are funded by clinical commissioning groups and run either by local mental health services or in partnership with acute trusts. Cognitive Neurology Clinics (CNCs) are increasingly provided by acute trusts and target younger populations with potential young onset dementias, and are more likely to see patients with FCD. As access to these services has improved, an increasing proportion of attendees are being diagnosed with FCD.[6] To date, the population prevalence of FCD has not been studied. However, a recent systematic review described 56 studies which demonstrated a high prevalence of cognitive symptoms in community populations (30% in 245,654 subjects).[3] The same review reported that 24% of DMCs attendees may have FCD, or one of its synonyms (range: 12–56%).

Those with FCD seeking referral to memory clinics have elevated levels of distress, depression, and anxiety.[3] Their symptoms persist and adversely impact employment status and activities of daily living (ADLs). Schmidtke and colleagues [7] found that cognitive symptoms persisted in 85% of those followed-up for an average of 20 months. One of their cohort (2.1%) went on to develop dementia, in keeping with the rate of revision of functional diagnoses and

For peer review only - http://bmjopen.bmj.com/site/about/guidelines.xhtml

neurological conditions generally.[8] Hence, FCD should not be regarded as the precursor for an inevitable dementia.

It is unclear how to help this group of patients. A recent survey of DMCs found 73% immediately discharged them to primary care and treatments offered ranged from simple reassurance to referral to a community mental health team.[9] As they receive little explanation for their symptoms, patients are liable to present to their GP requesting further referrals and investigations, with the potential for iatrogenic harm and unnecessary healthcare costs.

There is now preliminary evidence that strategies focused on expectations, cognitive restructuring and psychoeducation can be helpful.[10, 11] A recent meta-analysis of treatment studies to date found that group interventions involving both cognitive-training and expectancy-modification significantly improved psychological well-being. While expectancy-change interventions had little effect on objective measures of cognitive functioning, cognitive-training was associated with small, clinically insignificant improvements in tasks related to the training ones, with no generalisation to daily life.[11] No adverse events were described.

A rare, high quality RCT included in the meta-analysis involved 18 patients receiving 13 sessions of group cognitive-behavioural therapy (CBT) intended to change participants' beliefs and expectations.[10] Patients in the treatment group had significantly better memory-related self-efficacy than the controls at the end of the intervention and at 6-month follow-up (n=18). However, such interventions are not routinely provided by diagnostic memory, cognitive neurology, or neuropsychiatry clinics, where these patients are most likely to be seen. Furthermore, a 13-session intervention is resource intensive and unfeasible within DMCs and CNCs. Increasing Access to Psychological Therapies (IAPT) services are unlikely to offer any intervention beyond support for any co-morbid anxiety or depressive disorder.

St George's Hospital in South London, in collaboration with South West London and St George's NHS Mental Health Trust, is a centre of excellence for functional neurological disorders. We have developed a five-session group treatment based on the third-wave

For peer review only

cognitive behaviour therapy known as Acceptance and Commitment Therapy (ACT). This intervention focuses on changing a person's relationship with their thoughts and feelings. It makes use of mindfulness and acceptance processes and increases values-driven behaviour.[12] ACT considers psychological inflexibility – behaviour that is driven by attempts to excessively control internal experiences (such as difficult thoughts and feelings – as a source of emotional distress; hence, its aim is to enhance psychological flexibility.[13] Psychological flexibility is defined as "the ability to contact the present moment more fully as a conscious human being, and to change or persist in behaviour when doing so serves valued ends"[14] while psychological inflexibility is regarded a trans-diagnostic process common to numerous psychopathological states.[15]

Perceived threat is thought to be a maintenance factor for FND. Symptoms, such as cognitive failures in the case of FCD, are experienced as threatening, which causes hypervigilance and autonomic arousal. Symptoms are therefore experienced in a "top-down" manner - influenced by cognitive and neurobiological processes of expectations and predictions of illness.[5, 16] Improvement with ACT occurs through six key processes that can be grouped as either "mindfulness and acceptance processes" or "commitment and behaviour change processes".[12] Consequently, ACT aims to change a person's relationship with their internal experiences, increasing psychological flexibility and altering the top-down expectations by facilitating bottom-up processes, e.g., enhancing connection with direct experiences through mindfulness practices.

ACT's efficacy across a range of conditions has been demonstrated by several RCTs and meta-analyses. A recent review of 20 meta-analyses found it to be superior to inactive controls, treatment as usual (TAU), and active interventions (excluding CBT), with effect sizes ranging from small to medium.[17] There is evidence that ACT effectively reduces distress and disability in chronic pain [18] and long-term medical conditions [19] while it has also been recommended for the treatment of functional neurological disorders generally.[13, 20] ACT can be delivered in one-to-one or group formats. It is feasible and acceptable to deliver to patients with psychosis in a brief group format [21] and can be effectively delivered online through guided and unguided modules of learning.[22]

For peer review only

Unpublished initial pilot data suggested improvement in measures of quality of life and decreased psychological distress in four cohorts of patients who have received our brief ACT intervention. Following this, we were able to secure funding for a feasibility study from the National Institute for Health and Care Research (NIHR; grant number NIHR202743). We now aim to study the feasibility of delivering a randomised controlled trial of ACT for FCD as an online group intervention and compare this against current treatment as usual (TAU). We also aim to further refine the ACT intervention over the course of the study so it can be adapted and manualised for a future definitive randomised controlled trial.

Study objectives

The feasibility study aims to investigate:

- The willingness of clinicians in local services commissioned to assess patients with cognitive complaints to refer patients diagnosed with FCD into the study
- The willingness of patients with FCD to consent to the trial and be randomised to ACT+TAU vs TAU
- Acceptability of the online group ACT intervention
- Appropriateness and acceptability of clinical outcome measures
- Completion rates for outcome measures at the various time points and rate of adherence to the ACT intervention
- Fidelity of intervention
- Time needed to collect and analyse data
- Healthcare utilisation pre- and post-intervention
- Signal of efficacy in clinical outcomes

For peer review only

Methods

Trial design

ACT4FCD is a parallel-group, single-blind RCT, designed to assesses the feasibility of delivering a trial of a brief online group Acceptance and Commitment Therapy (ACT) and comparing it against the current standard intervention (Treatment As Usual (TAU)). Participants are assessed at baseline and again at 8 weeks, 16 weeks, and 26 weeks. In addition, the study aims to collect data on health utilisation before and after the intervention and has an embedded qualitative study of lived experience of FCD and the ACT intervention. This study adheres to the Standard Protocol Items Recommendations for Interventional Trials (SPIRIT; see Appendix 1).

Recruiting sites and participants

We are recruiting participants from Diagnostic Memory Clinics (DMCs), Neuropsychiatry Clinics (NPCs) Clinical Neurology Clinic (CNCs) in London. Recruitment began on 07/11/2022. These different clinics assess a spectrum of patients with cognitive symptoms. DMCs tend to see an older population while younger patients are more often referred to the NPC and CNC. If we are not successful recruiting participants from these sites alone then we will review within the trial management group and consider adding additional recruiting sites in nearby specialist clinics.

Clinicians in the recruiting clinics will refer potentially suitable patients to the study team and deliver the "treatment as usual" (TAU) intervention as part of routine clinical care. Potential participants will provide verbal consent to being contacted by the research team. Following this, they will receive the participant information sheet (see Appendix 2), including dates of the ACT groups, and the informed consent form (see Appendix 3) and given at least 24 hours to consider these. The consent form will also record whether participants are willing to be contacted about involvement in a parallel qualitative study, described below. Potential participants will be informed that choosing not to take part will not impact their medical treatment with any service. Once consent is given, the research assistant contacts the potential participant to complete a screening interview against the inclusion/ exclusion criteria (see Figure 1. for Study Flow Chart). The inclusion/ exclusion criteria are:

For peer review only

Inclusion criteria

- An established diagnosis of FCD made in DMC/CNC/NPC and confirmed by research team from review of clinical notes and examination findings
- Aged 18 or over
- Capacity to provide written informed consent

Exclusion criteria

- Disabling cognitive symptoms in the context of a primary psychiatric disorder (e.g. depression, severe generalised anxiety disorder, PTSD, bipolar affective disorder, schizophrenia).
- Greater than mild-moderate depressive or anxiety disorders (PHQ9 score ≥ 15 and/or GAD7 score ≥ 15)
- At "medium" or "high" risk of deliberate self-harm and/or suicide (based on clinical assessment)
- Another predominant functional disorder (e.g., functional seizures)*

*Co-morbid functional diagnosis is acceptable so long as those symptoms do not dominate the clinical picture.

For peer review only

- Diagnosis of dementia
- Diagnosis of learning disability
- Insufficient command of English to engage in conversation without an interpreter (as this would not be compatible with the online ACT intervention)

Primary outcome measures and progression criteria

The feasibility study is primarily gathering data on the feasibility of conducting a future definitive RCT. The primary outcome measures (and progression criteria) are:

- Rate of successful recruitment (≥70% intended participants recruited)
- Rate of successful adherence (≥75% in ACT + TAU attend four or more sessions)
- Acceptability of the ACT intervention (qualitative interview themes and majority (≥75%) satisfied/very satisfied on 5-point Likert scale)
- Signal of efficacy (based on increased psychological flexibility following intervention)

Randomisation

Participants will be randomised into ACT (plus TAU) or TAU using a simple block randomisation procedure (with randomly permuted block sizes of 2 and 4). Randomisation will be carried out via the online service Sealed Envelope by the Trial Manager, who will then inform participants of their arm allocation.

Blinding

Given the nature of the intervention, it is not possible to blind the participants to their intervention or those responsible for delivering the intervention (NP, SC, AD). The research assistant collecting the outcome data and the statistician will remain blind to treatment allocation (single-bind trial). Clinical outcome measures will be completed again at 8 weeks (T1), 16 weeks (T2), and 26 weeks (T3). Unblinding will be allowed only in case of an Serious Adverse Event (eg, resulting in death).

Secondary (clinical) outcome measures

Those who are deemed eligible to participate in the trial will be sent an individualised weblink to complete baseline (T0) clinical outcome measures. These can be completed on paper if the

For peer review only

participant prefers. The paper forms will be returned to the research assistant for inputting into the online database and then securely destroyed.

Clinical outcome measures will be completed at baseline (T0) and at 8 weeks (T1), 16 weeks (T2), and 26 weeks (T3). The proposed clinical outcome measures and the domains being measured include:

Health-related quality of life/functioning

- World Health Organisation Disability Assessment Schedule (WHODAS 2.0) [23]
- EuroQol 5-Dimension, 5-Level Health Scale (EQ-5D-5L) [24]
- ICEpop CAPability measure for Adults (ICECAP-A) [25]

Subjective cognitive symptoms

- Multifactorial Memory Questionnaire (MMQ) [26]

Depression and anxiety

- 9-question Patient Health Questionnaire (PHQ-9) [27]
- Generalized Anxiety Disorder 7 (GAD-7) [28]

ACT specific measure of change

- Acceptance and Action Questionnaire II (AAQ-II) [29] is a measure of psychological flexibility/inflexibility widely used in ACT. This would potentially be the primary outcome measure in a future definitive RCT.

Service utilisation and other cost variables

The Adult Service Use Schedule (AD-SUS) [30] is a self-report service use questionnaire completed by the study participant in interview with a trained researcher. The AD-SUS was developed by the study economist for previous work in similar populations and has been adapted for the needs of this study so that participants can complete this without assistance.

Improvement

- Clinical Global Impression – Improvement Scale (CGI-I), single item, participant rated

For peer review only

Measure of satisfaction

- Satisfaction rating of treatment (single-item, 5-point Likert scale)

The various research activities and outcome measures and the time points when they are collected are listed in Table 1.

Table 1. Protocol schedule of procedures for the ACT4FCD study

	Screening	Baseline (T0)	2 months (T1)	4 Months (T2)	6 months (T3)
**Enrolment**					
Consent to contact obtained by clinical staff in DMC, CNC, or NPC	x				
Informed consent	x				
Contacted by RA to arrange screening interview	x				
**Screening**					
Diagnostic criteria for FCD	x				
Risk Assessment	x				
Demographics	x				
Co-morbidities	x				
Medications	x				
PHQ-9	x				
GAD-7	x				
Randomisation allocation		x			
**Interventions**		ACT+TAU	Intervention completed		
**Assessments**					
AAQ-II		x	x	x	X
MMQ		x	x	x	X
WHODAS 2.0		x	x	x	X
EQ-5F-5L		x	x	x	X
PHQ-9		x	x	x	X
GAD-7		x	x	x	X
AD-SUS		x			X
ICECAP-A		x	x	x	X
CGI-I					X

Likert 5-point satisfaction scale				x	
AEs/SAEs		x	x	x	X
**Qualitative Interviews**					
Informed consent for qualitative interview (select participants)		x	x		
Qualitative interviews with select sample of participants		x	x		

Participant payment

Participants will receive a non-contingent payment of £25 for taking part in the trial. To aid retention, participants will receive £10 at each time point clinical outcome measures are fully completed. Those who take part in the optional qualitative interviews (see below) will also receive an additional £20 incentive.

The ACT intervention

The group consists of psychoeducation about normal memory functioning, including the roles of attention and normal patterns of forgetting. The psychoeducation element aims to reduce the threat of cognitive symptoms. In line with the ACT model, the aim is to increase psychological flexibility (our proposed primary clinical outcome measure for a future RCT; Acceptance and Action Questionnaire II (AAQ-II)) in response to the symptoms and associated thoughts and feelings.

The concept of "secondary suffering" is introduced. [32] It is suggested that attempts to control cognitive "failures" leads to additional suffering, such as ruminations, negative predictions, and avoidance. It is explicitly stated that the intervention does not aim to reduce "primary suffering" (the cognitive symptoms), although it is possible that improvements *may* occur if secondary suffering is reduced. Brief mindfulness practices are incorporated in the

For peer review only

group to facilitate "bottom up" processing, acceptance, and more neutral interpretations of unwanted experiences. Value-based goals are identified throughout the sessions in order to shift away from avoidance-based behaviour and the focus on cognitive symptoms.

Participants will be given the time of all ACT sessions prior to randomisation and are asked to consent only if able to attend should they be randomised to the active intervention. They will sent an email with link and diary invite for all the sessions the week before the first session and follow up reminder calls/emails each week to maximise attendance and engagement despite their memory problems. If a participant fails to attend a session, they will receive a follow up call from the research assistant to ensure they had the time and link and to enquire about future attendance.

The intervention protocol (see Appendix 4) will be amended in response to specific feedback received during the contemporaneous qualitative interviews which are designed to explore the participants experience of and satisfaction with the groups.

The intervention group will also receive TAU, as below.

Treatment as usual

Treatment as usual (TAU) was selected as the fairest comparison, given that is what most patients in the UK currently receive within mental health and cognitive neurology settings. It consists of an explanation of the FCD diagnosis, provision of additional information about the diagnosis and underlying factors (such as medications, chronic pain, or poor sleep hygiene; information: https://www.neurosymptoms.org/en/symptoms/fnd-symptoms/functional-cognitive-symptoms), and signposting to local psychological services in primary care (Increasing Access to Psychological Therapies (IAPT)) for appropriate treatment, such as CBT, when co-morbid anxiety and/or depression have been identified. TAU will be delivered as part of routine clinical care by the recruiting service.

Withdrawal and non-adherence

Participants who do not attend out of the SF-GAPI modules or assessments are not replaced. Disclosed reasons for withdrawal, non-adherence or loss to follow-up are reported.

For peer review only

Sample size

This is a feasibility trial; as such, a power calculation is neither possible nor necessary. Rather, the sample size is pragmatic. Target recruitment is 48 patients in total (24 in each treatment arm), which provides sufficiently reliable estimates of feasibility outcomes such as recruitment, adherence and retention rates to inform a fully powered RCT. For example, assuming 70% of those approached consent to participate in the trial, the 95% confidence interval for adherence rate would have width of 13.0%. For those in the treatment arm, the 95% confidence interval for an intervention adherence rate of 80% would have width of 16.0%. A sample size of 48 is also consistent with those recommended for pilot and feasibility studies to provide adequate data and precision of means and variances (*n* between 24 and 50). [33, 34, 35] If it proves challenging to recruit participants via these routes alone then we will approach colleagues at other specialist services in London who see patients with FCD to increase our recruitment pool.

Statistical analysis plan

A fully documented statistical analysis plan, centred on describing key process measures to decide if a definitive trial is feasible, will be prepared by the statistician (SAP), formally agreed with co-investigators and the Trial Steering Committee prior to data collection being completed. Participant throughput will be summarized in an extended Consolidated Standards of Reporting Trials (CONSORT) diagram.[36] The CONSORT flow chart will be used to present descriptive data on trial referral, enrolment, intervention allocation, adherence, and retention and to document any deviations from protocol.

Feasibility outcomes will be summarised using descriptive statistics, with 95% confidence intervals (CIs) provided to permit assumptions when planning a future definitive trial. Data relating to referral, screening and enrolment, and recruitment logs will be used to produce accurate estimates of eligibility, consent, and recruitment rates.

Treatment assignation, intervention adherence (e.g., ACT session attendance) and satisfaction of care data will be used to contribute to the evaluation of the acceptability of randomisation and allocated intervention/treatment arms. At each time point, the time taken

For peer review only

(per participant) to complete trial measures will be recorded. Retention rates will also be estimated for each of the patient-reported/clinical outcome measures, with consideration given to differential dropout between the arms of the trial to identify potential (attrition) bias in treatment completion and/or data collection. All feasibility outcomes will be compared with full-trial progression criteria.

Baseline characteristics will be reported according to treatment arm. Continuous variables will be reported as mean (standard deviation (SD)) if normally distributed or median (inter-quartile range) if non-normal, while categorical variables will be presented as frequency (%). Subsequent analyses will summarise the proposed patient-reported and clinical outcomes (e.g., psychological flexibility, subjective memory function, quality of life, and depression/anxiety measures) at each time point for each trial arm using appropriate descriptive statistics (e.g., group mean, SD). To provide an indication of potential changes in scores/frequencies between the four time points, linear/logistic mixed effects regression models will be employed performed on an intention-to-treat (ITT) basis (accounting for data assumed to be missing at random). These random intercept (mixed) models will include intervention group, time, and intervention group by time interaction. There will be no emphasis on hypothesis testing, however, which is reserved for the future main trial. Rather, pre-to-post-intervention standardised effect sizes (Hedges' g, relative risk) will be computed (SDs will be computed from estimated model standard errors) with associated CIs calculated to explore imprecision around effect sizes.[37] Due to the small sample size, important covariates (e.g., baseline score on relevant measure, gender, age) may be included in models if the two arms happen to be highly imbalanced. ITT analyses will also be administered by imputing values for missing data using a conservative last observation carried forward (LOCF) procedure (given full sensitivity analysis testing of missing data assumptions is beyond the scope of a feasibility study). Exploratory analyses using mixed effect models will examine the rate of change in intervention and control groups on outcome measures across four time-points, adjusting for relevant baseline scores and variables of interest (anxiety, depression, subjective evaluation of memory), and investigate changes on a per-protocol basis (focused on intervention adherence; i.e., including only participants who attended at least 4 sessions and with post-treatment data).

For peer review only

The qualitative study

A sub-sample of participants in both arms of the trial will be invited for in-depth semi-structured interviews over the course of the intervention period. A sampling framework will be used that ensures participants are included that are representative of the sociodemographic characteristics and clinical profile. Interviews at baseline (T0) will focus on the experience of living with FCD and subsequent interviews (at T1), not necessarily with the same participants, will explore their views on the ACT intervention. These interviews will focus on the acceptability, number, and frequency of ACT sessions, ways of optimising engagement, perceived benefits/limitations of the intervention, and any recommendations for improvement of the components and/or content of the intervention.

Interview schedules will be coproduced by the PPI representatives and the research team. One-to-one, in-depth interviews will be carried out via Teams by the research assistant, recorded, and transcribed, and the transcripts cross-checked against the original recordings to ensure accuracy. The analysis will be led by the qualitative study expert and research assistant using reflexive thematic analysis [38] aided by NVivo12 software. The results of the T1 interviews will be utilised to optimise the intervention during the feasibility study with the aim of developing a formalised intervention to be used in a future RCT.

Data management

All data will be inputted by the participants themselves on to the trial database on REDCap and backed up weekly on a secure server. The electronic Trial Master File will be backed up weekly on an additional encrypted hard drive. No paper copies will be stored. Please refer to Data Management Plan (see Appendix 5) for details regarding confidentiality, data collection, data handling, and data transfer. The data collection and management will be in line with GDPR Data Protection Act (2018) and SWLSTG's Information Governance.

For peer review only

Patient and public involvement

The research design has been informed by our PPI representatives who were recruited from earlier pilots of the intervention conducted within the SWLSTG Neuropsychiatry Service. They assisted in the development of the intervention, study methodology, and review of clinical outcome measures. Two PPI participants (SB, MS) have been recruited to the Trial Steering Committee and are funded to assist with reviewing study materials, writing, and developing patient information leaflets, producing the semi-structured interview schedules for the qualitative study, and ensuring proper conduct of the study. We will document PPI activity over the course of the study in order to accurately assess where and how the lived experience perspective has been used and its impact on the research process and findings. All PPI representatives have lived experience of FCD and the proposed ACT intervention.

Serious adverse events

All adverse events (AEs) and serious adverse events (SAEs) reported spontaneously by participants or observed by researchers will be recorded and reported to the Trail Manager (TM) and Chief Investigator (CI). Urgent actions concerning participant and staff safety, communication with others, and clinical care will be immediately addressed by the CI and reported to the Trial Management Group (TMG). A summary of (S)AEs will be presented at each Trial Management Group (TMG) and Trial Steering Committee (TSC) meeting. AEs will be categorised for severity and seriousness by the TM and CI. SAEs will be further reviewed for relatedness to trial procedures and unexpectedness by the CI initially, and additionally by the chair of the TSC.

For peer review only

Ethics and dissemination

Ethics approval was sought from the South East Scotland Research Ethics Committee (REC) 02 and a favourable opinion was received on 30th September 2022 (REC reference: 22/SS/0059). Any amendments to the protocol will be agreed with the REC before being implemented and then amended on the ISCTRN Registry. The findings of the study will be published on an open access journal once the full trial has been completed. A data monitoring committee was not deemed necessary as participants are adverse effects are not expected in either of the randomisation groups.

For peer review only

Author contributions

NP, SC, and ME conceived the study. NP, SC, ME, NM, JS, and RH, developed and finalised the study design. JS provided statistical expertise in the clinical trial design and NM provided qualitative research expertise in the qualitative study design. BB contributed to the design of the health economics analysis. SB, MS, and KT provided expert by experience expertise. All authors contributed to refinement of the study protocol. NP drafted the manuscript. All authors provided critical revisions to the manuscript and approved the final manuscript. NP is grant holder. NP, SC, SV, JS, ME, RH, and BB will have access to the final trial dataset. The authors intend to share deidentified individual clinical trial participant-level data after publication of results with researchers who provide a methodologically sound proposal. Requests can be sent to norman.poole@gmail.com

Study sponsor details

South West London & St George's Mental Health NHS Trust (SWLSTG)

Sponsor contact:

Tania West

R&D Governance & Clinical Trials Manager

South West London & St George's Mental Health NHS Trust

Research & Development, Newton Building (1st Floor)

Springfield University Hospital

Glenburnie Road

London SW17 7DJ

Email: researchdevelopment@swlstg.nhs.uk

For peer review only

Sponsor roles and responsibilities

SWLSTG Mental Health NHS Trust is not involved in any aspect of the study design and management, or in its data collection, analysis or interpretation. The Sponsor is responsible for providing the research team with adequate arrangements to successfully complete the trial, and to monitor its compliance with ethical guidelines and legislation. SWLSTG Mental Health NHS Trust is the data controller for the study and all data shall return to the Sponsor at the end of the trial. A collaboration agreement is being finalised with King's College London, where a Co-Investigator and a Trial Statistician are based. No Data Sharing Agreements are required.

Funding statement

This study is funded by the NIHR Research for Patient Benefit (RfPB) Programme (NIHR202743) and supported by the NIHR Clinical Research Network. The views expressed are those of the author(s) and not necessarily those of the NIHR or the Department of Health and Social Care.

Funder roles and responsibilities

NIHR is not involved in any aspect of the study design and management, or in its data collection, analysis or interpretation. Core publications (including, but not limited to, research protocol and results) were agreed with NIHR before the beginning of the trial, and NIHR will be informed of any additional publications. NIHR will also be acknowledged in any public dissemination of the research, including peer-reviewed articles, conference presentations and patient involvement activities.

Trial steering committee roles and responsibilities

The TSC will oversee the study on behalf of the of the trial Sponsor and Funder and ensure that the study is conducted within appropriate NHS and professional ethical guidelines. It will provide advice on all appropriate aspects of the project; will oversee progress of the trial, adherence to the protocol, participant safety and the consideration of new information of

For peer review only

relevance to the research question; will ensure the rights, safety and well-being of the participants are given the most important considerations; will ensure appropriate ethical and other approvals are obtained in line with the project plan; will agree proposals for substantial protocol amendments and provide advice to the Sponsor and Funder regarding approvals of such amendments. It will comprise the CI (NP), TM (SV), independent Chair (Professor Michael Kopelman), independent member (Dr Laure McWhirter), sponsor representative, and two independent PPI members.

Trial management group roles and responsibilities

The TMG is composed of AD, BB, JS, ME, NM, NP, RH, SB, SC, SV. The agreed terms of reference are:

1. To review and provide feedback on any amendment of the study protocol, outcome measures and recruitment documents
2. To support the progress of the trial towards its interim and overall objectives and against the study timescale
3. To agree on a trial monitoring plan
4. To advise on participant recruitment and retention
5. To facilitate data analysis
6. To help troubleshoot challenges to the timely completion of the trial
7. To review the study outcomes
8. To discuss future developments of the study
9. To report to the Trial Steering Committee (TSC)

Data availability statement

The data that support the findings of this study will be openly available in ISRCTN Registry at https://doi.org/10.1186/ISRCTN12939037

For peer review only

Competing interests statement

NP – No competing interests

SC – No competing interests

SV – No competing interests

AD – No competing interests

NM – No competing interests

JS – No competing interests

MS – No competing interests

SB – No competing interests

KT – No competing interests

ME – No competing interests

RH – No competing interests

For peer review only

References

1. Ball HA, McWhirter L, Ballard C, et al. Functional cognitive disorder: dementia's blind spot. *Brain* 2020;143(10):2895-2903. doi.org/10.1093/brain/awaa224

2. Poole N, Cope S, Bailey C, Isaacs J. Functional cognitive disorders: Identification and management. *BJPsych Advances* 2019; **25(6)**: 342-350. doi:10.1192/bja.2019.38

3. McWhirter L, Ritchie C, Stone J, Carson A. Functional cognitive disorders - a systematic review. *Lancet Psychiatry*, 2020;**7(2)**: 191-207. doi: 10.1016/S2215-0366(19)30405-5

4. Teodoro T, Edwards M, Isaacs J. A unifying theory for cognitive abnormalities in functional neurological disorders, fibromyalgia and chronic fatigue syndrome: systematic review. *J Neurol Neurosurg Psychiatry* 2018;**89**:1-12. doi:10.1136/jnnp-2017-317823

5. Edwards MJ, Adams RA, Brown H, et al. A Bayesian account of 'hysteria'. *Brain* 2012;**135(11)**:495–512.

6. Larner AJ Impact of the National Dementia Strategy in a neurology-led memory clinic: 5-year data. *Clinical Medicine* 2014;**14**:216. doi: 10.7861/clinmedicine.14-2-216.

7. Schmidtke K, Pohlmann S, Metternich B. The syndrome of functional memory disorder: definition, etiology, and natural course. *Am J Geriatr Psychiatry* 2008;**16**:981–8. doi: 10.1097/JGP.0b013e318187ddf9

8. Stone J, Smyth R, Carson A, et al. Systematic review of misdiagnosis of conversion symptoms and "hysteria". *Br Med J* 2005;**331**:989. doi: 10.1136/bmj.38628.466898.55

9. Bailey C, Bell SM, Blackburn DM. How the UK describes functional memory symptoms. *Psychogeriatrics* 2017;**17**: 336–7.

10. Metternich B, Kosch D, Kriston L, et al. The effects of nonpharmacological interventions on subjective memory complaints: a systematic review and meta-analysis. *Psychotherapy and Psychosomatics* 2010;**79**:6–19. doi: 10.1159/000254901

11. Bhome R, Berry AJ, Huntley JD, et al. Interventions for subjective cognitive decline: systematic review and meta-analysis. *BMJ Open* 2018;**8**:e021610. doi: 10.1136/bmjopen-2018-021610

12. Hayes SC, Luoma JB, Bond FW, et al. Acceptance and commitment therapy: Model, processes and outcomes. *Behavior Research and Therapy* 2006;**44**:1–25. doi: 10.1016/j.brat.2005.06.006

For peer review only

13. Cope SR, Poole N, Agrawal N. Treating functional non-epileptic attacks - Should we consider acceptance and commitment therapy? *Epilepsy Behav* 2017;**73**:197-203. doi: 10.1016/j.yebeh.2017.06.003

14. Hayes SC, Luoma JB, Bond FW, Masuda A, Lillis J. Acceptance and commitment therapy: model, processes and outcomes. *Behav Res Ther* 2006;44:1–25. doi: 10.1016/j.brat.2005.06.006.

15. Levin ME, MacLane C, Daflos S, et al. Examining psychological inflexibility as a transdiagnostic process across psychological disorders. *Journal of Contextual Behavioral* Science 2014;**3**:155–63. doi: 10.1016/j.jcbs.2014.06.003

16. Brown RJ, Reuber, M. Towards an integrative theory of psychogenic non-epileptic seizures (PNES). *Clinical Psychology Review* 2016;47:55–70. doi: 10.1016/j.cpr.2016.06.003.

17. Gloster AT, Walder N, Levin ME, et al. The empirical status of acceptance and commitment therapy: A review of meta-analyses. *Journal of Contextual Behavioral Science* 2020;**18**:181-192. doi: 10.1016/j.jcbs.2020.09.009.

18. Du S, Dong J, Jin S, Zhang H, Zhang Y. Acceptance and Commitment Therapy for chronic pain on functioning: A systematic review of randomized controlled trials. *Neurosci Biobehav Rev* 2021;**131**:59-76. doi: 10.1016/j.neubiorev.2021.09.022

19. Graham CD, Gouick J, Krahé C, Gillanders D. A systematic review of the use of Acceptance and Commitment Therapy (ACT) in chronic disease and long-term conditions. *Clinical Psychology Review*, 2016;**46**:46-58. doi: 10.1016/j.cpr.2016.04.009

20. Graham CD, O'Hara DJ, Kemp S. A case series of Acceptance and Commitment Therapy (ACT) for reducing symptom interference in functional neurological disorders. *Clinical Psychology and Psychotherapy*, 2018;25 (3):489-96. doi: 10.1002/cpp.2174

21. Johns LC, Oliver JE, Khondoker M, et al. The feasibility and acceptability of a brief Acceptance and Commitment Therapy (ACT) group intervention for people with psychosis: The 'ACT for life' study. *J Behav Ther Exper Psychiatry* 2016;50:257-63. doi: 10.1016/j.jbtep.2015.10.001

22. Thompson EM, Destree L, Albertella L, Fontenelle LF. Internet-Based Acceptance and Commitment Therapy: A Transdiagnostic Systematic Review and Meta-Analysis for Mental Health Outcomes. *Behavior Therapy* 2021;52(2):492-507. doi: 10.1016/j.beth.2020.07.002

For peer review only

23. World Health Organisation (WHO) (2010). Measuring Health and Disability: Manual for WHO Disability Assessment Schedule (WHODAS 2.0). Edited by TB Üstün, N Kostanjsek, S Chatterji, J Rehm. Geneva (CH): World Health Organization.

24. Herdman M, Gudex C, Lloyd A, Janssen M, Kind P, Parkin D, Bonsel G, Badia X. Development and preliminary testing of the new five-level version of EQ-5D (EQ-5D-5L). Qual Life Res. 2011 Dec;20(10):1727-36. doi: 10.1007/s11136-011-9903-x.

25. Al-Janabi H, Flynn T, Coast J. (2012). Development of a self-report measure of capability wellbeing for adults: the ICECAP-A. Quality of Life Research 21: 167-76. doi: 10.1007/s11136-011-9927-2.

26. Troyer AK, Rich JB (2002). Psychometric properties of a new metamemory questionnaire for older adults. Journal of Gerontology, 57(1), 19-27. doi: 10.1093/geronb/57.1.p19.

27. Kroenke K, Spitzer RL, Williams JB (2001) The PHQ-9: validity of a brief depression severity measure. J Gen Intern Med, 16(9): 606-13. doi: 10.1046/j.1525-1497.2001.016009606.x

28. Spitzer RL, Kroenke K, Williams JBW, Lowe B (2006) A brief measure for assessing generalized anxiety disorder. Arch Inern Med, 166: 1092-7. doi: 10.1001/archinte.166.10.1092.

29. Bond FW, Hayes SC, Baer RA, Carpenter KM, Guenole N, Orcutt HK, Waltz T, Zettle RD. Preliminary psychometric properties of the Acceptance and Action Questionnaire-II: a revised measure of psychological inflexibility and experiential avoidance. Behav Ther. 2011;42(4):676-88. doi: 10.1016/j.beth.2011.03.007.

30. Tyrer P, Cooper S, Salkovskis P, Tyrer H, Crawford M, Byford S, Dupont S, Finnis S, Green J, McLaren E, Murphy D, Reid S, Smith G, Wang D, Warwick H, Petkova H, Barrett B. Clinical and cost-effectiveness of cognitive behaviour therapy for health anxiety in medical patients: a multicentre randomised controlled trial. Lancet. 2014 18;383(9913):219-25. doi: 10.1016/S0140-6736(13)61905-4.

31. Guy W. Clinical Global Impressions (CGI) Scale, Modified. In Rush, John A.; Task Force for the Handbook of Psychiatric Measures (eds.). *Handbook of Psychiatric Measures* (1st ed.). Washington, DC: American Psychiatric Association. 2000. ISBN 978-0-89042-415-5. OCLC 43483679.

32. Buhrman M, Skoglund A, Husell J, Bergström K, Toet al. Guided Internet-Delivered Acceptance and Commitment Therapy for Chronic Pain Patients: A Randomized Controlled

For peer review only

Trial. *Behaviour Research and Therapy* 2013;51(6): 307–15. doi:10.1016/J.BRAT.2013.02.010.

33. Julius SA. Sample size of 12 per group rule of thumb for a pilot study. Pharm Stat, 2005;4:287-91. doi.org/10.1002/pst.185.

34. Lancaster GA, Dodd S, Williamson PR. Design and analysis of pilot studies: recommendations for good practice. J Eval Clin Practice 2004;10:307-12. doi: 10.1111/j..2002.384.doc.x.

35. Sim J, Lewis M. The size of a pilot study for a clinical trial should be calculated in relation to considerations of precision and efficiency. J Clin Epidemiol 2012;65:301-8. doi: 10.1016/j.jclinepi.2011.07.011.

36. Eldridge SM, Chan CL, Campbell MJ, Bond CM, Hopewell S, Thabane L et al. CONSORT 2010 statement: extension to randomised pilot and feasibility trials *BMJ* 2016;355:i5239. doi:10.1136/bmj.i5239

37. Durlak JA. How to select, calculate, and interpret effect sizes. *Journal of Pediatric Psychology* 2009; 34(9): 917-28. doi: 10.1093/jpepsy/jsp004.

38. Braun V, Clarke V. Using thematic analysis in psychology. *Qual Res Psychol* 2006; 3: 77–101.

For peer review only

Figure 1. Study Flow Chart

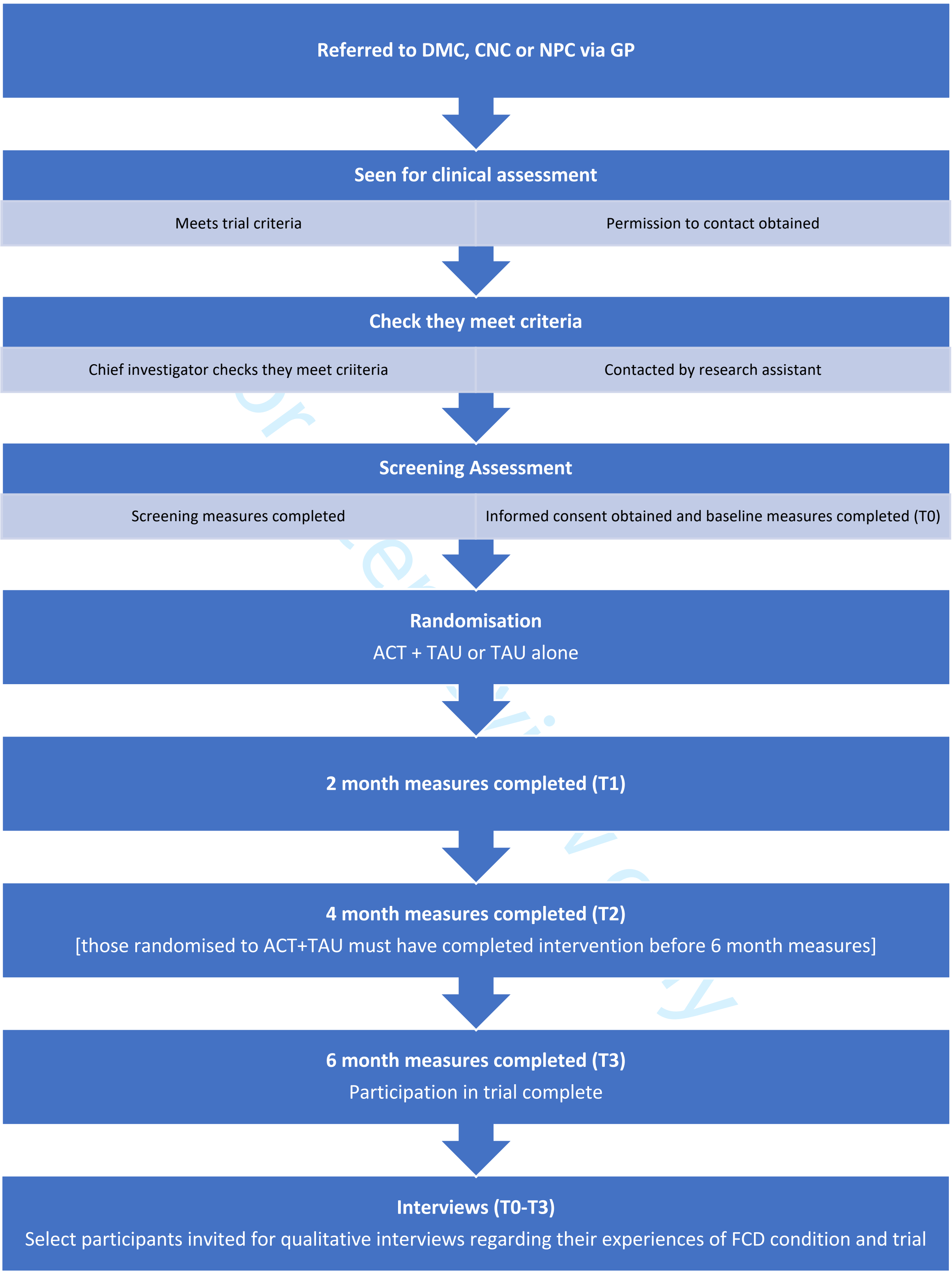

Reporting checklist for protocol of a clinical trial.

Based on the SPIRIT guidelines.

Instructions to authors

Complete this checklist by entering the page numbers from your manuscript where readers will find each of the items listed below.

Your article may not currently address all the items on the checklist. Please modify your text to include the missing information. If you are certain that an item does not apply, please write "n/a" and provide a short explanation.

Upload your completed checklist as an extra file when you submit to a journal.

In your methods section, say that you used the SPIRITreporting guidelines, and cite them as:

Chan A-W, Tetzlaff JM, Gøtzsche PC, Altman DG, Mann H, Berlin J, Dickersin K, Hróbjartsson A, Schulz KF, Parulekar WR, Krleža-Jerić K, Laupacis A, Moher D. SPIRIT 2013 Explanation and Elaboration: Guidance for protocols of clinical trials. BMJ. 2013;346:e7586

		Reporting Item	Page Number
**Administrative information**			
Title	#1	Descriptive title identifying the study design, population, interventions, and, if applicable, trial acronym	1
Trial registration	#2a	Trial identifier and registry name. If not yet registered, name of intended registry	4
Trial registration: data set	#2b	All items from the World Health Organization Trial Registration Data Set	
Protocol version	#3	Date and version identifier	2
Funding	#4	Sources and types of financial, material, and other support	23

For peer review only

Roles and responsibilities: contributorship	#5a	Names, affiliations, and roles of protocol contributors	22
Roles and responsibilities: sponsor contact information	#5b	Name and contact information for the trial sponsor	22
Roles and responsibilities: sponsor and funder	#5c	Role of study sponsor and funders, if any, in study design; collection, management, analysis, and interpretation of data; writing of the report; and the decision to submit the report for publication, including whether they will have ultimate authority over any of these activities	22
Roles and responsibilities: committees	#5d	Composition, roles, and responsibilities of the coordinating centre, steering committee, endpoint adjudication committee, data management team, and other individuals or groups overseeing the trial, if applicable (see Item 21a for data monitoring committee)	23-24
**Introduction**			
Background and rationale	#6a	Description of research question and justification for undertaking the trial, including summary of relevant studies (published and unpublished) examining benefits and harms for each intervention	5-8
Background and rationale: choice of comparators	#6b	Explanation for choice of comparators	12-14
Objectives	#7	Specific objectives or hypotheses	8
Trial design	#8	Description of trial design including type of trial (eg, parallel group, crossover, factorial, single group), allocation ratio, and framework (eg, superiority, equivalence, non-inferiority, exploratory)	9

For peer review only

**Methods: Participants, interventions, and outcomes**			
Study setting	#9	Description of study settings (eg, community clinic, academic hospital) and list of countries where data will be collected. Reference to where list of study sites can be obtained	9
Eligibility criteria	#10	Inclusion and exclusion criteria for participants. If applicable, eligibility criteria for study centres and individuals who will perform the interventions (eg, surgeons, psychotherapists)	9-10
Interventions: description	#11a	Interventions for each group with sufficient detail to allow replication, including how and when they will be administered	12-14
Interventions: modifications	#11b	Criteria for discontinuing or modifying allocated interventions for a given trial participant (eg, drug dose change in response to harms, participant request, or improving / worsening disease)	n/a
Interventions: adherance	#11c	Strategies to improve adherence to intervention protocols, and any procedures for monitoring adherence (eg, drug tablet return; laboratory tests)	10 & 13
Interventions: concomitant care	#11d	Relevant concomitant care and interventions that are permitted or prohibited during the trial	13-14
Outcomes	#12	Primary, secondary, and other outcomes, including the specific measurement variable (eg, systolic blood pressure), analysis metric (eg, change from baseline, final value, time to event), method of aggregation (eg, median, proportion), and time point for each outcome. Explanation of the clinical relevance of chosen efficacy and harm outcomes is strongly recommended	10-12
Participant timeline	#13	Time schedule of enrolment, interventions (including any run-ins and washouts), assessments, and visits	19-20

For peer review only

		for participants. A schematic diagram is highly recommended (see Figure)	
Sample size	#14	Estimated number of participants needed to achieve study objectives and how it was determined, including clinical and statistical assumptions supporting any sample size calculations	14
Recruitment	#15	Strategies for achieving adequate participant enrolment to reach target sample size	9
**Methods: Assignment of interventions (for controlled trials)**			
Allocation: sequence generation	#16a	Method of generating the allocation sequence (eg, computer-generated random numbers), and list of any factors for stratification. To reduce predictability of a random sequence, details of any planned restriction (eg, blocking) should be provided in a separate document that is unavailable to those who enrol participants or assign interventions	10
Allocation concealment mechanism	#16b	Mechanism of implementing the allocation sequence (eg, central telephone; sequentially numbered, opaque, sealed envelopes), describing any steps to conceal the sequence until interventions are assigned	10
Allocation: implementation	#16c	Who will generate the allocation sequence, who will enrol participants, and who will assign participants to interventions	10 & 19
Blinding (masking)	#17a	Who will be blinded after assignment to interventions (eg, trial participants, care providers, outcome assessors, data analysts), and how	11
Blinding (masking): emergency unblinding	#17b	If blinded, circumstances under which unblinding is permissible, and procedure for revealing a participant's allocated intervention during the trial	11

For peer review only

**Methods: Data collection, management, and analysis**			
Data collection plan	#18a	Plans for assessment and collection of outcome, baseline, and other trial data, including any related processes to promote data quality (eg, duplicate measurements, training of assessors) and a description of study instruments (eg, questionnaires, laboratory tests) along with their reliability and validity, if known. Reference to where data collection forms can be found, if not in the protocol	11
Data collection plan: retention	#18b	Plans to promote participant retention and complete follow-up, including list of any outcome data to be collected for participants who discontinue or deviate from intervention protocols	12
Data management	#19	Plans for data entry, coding, security, and storage, including any related processes to promote data quality (eg, double data entry; range checks for data values). Reference to where details of data management procedures can be found, if not in the protocol	16
Statistics: outcomes	#20a	Statistical methods for analysing primary and secondary outcomes. Reference to where other details of the statistical analysis plan can be found, if not in the protocol	14-16
Statistics: additional analyses	#20b	Methods for any additional analyses (eg, subgroup and adjusted analyses)	14-16
Statistics: analysis population and missing data	#20c	Definition of analysis population relating to protocol non-adherence (eg, as randomised analysis), and any statistical methods to handle missing data (eg, multiple imputation)	14-16
**Methods: Monitoring**			

For peer review only

Data monitoring: formal committee	#21a	Composition of data monitoring committee (DMC); summary of its role and reporting structure; statement of whether it is independent from the sponsor and competing interests; and reference to where further details about its charter can be found, if not in the protocol. Alternatively, an explanation of why a DMC is not needed	18
Data monitoring: interim analysis	#21b	Description of any interim analyses and stopping guidelines, including who will have access to these interim results and make the final decision to terminate the trial	n/a
Harms	#22	Plans for collecting, assessing, reporting, and managing solicited and spontaneously reported adverse events and other unintended effects of trial interventions or trial conduct	17
Auditing	#23	Frequency and procedures for auditing trial conduct, if any, and whether the process will be independent from investigators and the sponsor	n/a
**Ethics and dissemination**			
Research ethics approval	#24	Plans for seeking research ethics committee / institutional review board (REC / IRB) approval	3
Protocol amendments	#25	Plans for communicating important protocol modifications (eg, changes to eligibility criteria, outcomes, analyses) to relevant parties (eg, investigators, REC / IRBs, trial participants, trial registries, journals, regulators)	18
Consent or assent	#26a	Who will obtain informed consent or assent from potential trial participants or authorised surrogates, and how (see Item 32)	9
Consent or assent: ancillary studies	#26b	Additional consent provisions for collection and use of participant data and biological specimens in ancillary studies, if applicable	n/a

For peer review only

Confidentiality	#27	How personal information about potential and enrolled participants will be collected, shared, and maintained in order to protect confidentiality before, during, and after the trial	16
Declaration of interests	#28	Financial and other competing interests for principal investigators for the overall trial and each study site	25
Data access	#29	Statement of who will have access to the final trial dataset, and disclosure of contractual agreements that limit such access for investigators	
Ancillary and post trial care	#30	Provisions, if any, for ancillary and post-trial care, and for compensation to those who suffer harm from trial participation	n/a
Dissemination policy: trial results	#31a	Plans for investigators and sponsor to communicate trial results to participants, healthcare professionals, the public, and other relevant groups (eg, via publication, reporting in results databases, or other data sharing arrangements), including any publication restrictions	18
Dissemination policy: authorship	#31b	Authorship eligibility guidelines and any intended use of professional writers	n/a
Dissemination policy: reproducible research	#31c	Plans, if any, for granting public access to the full protocol, participant-level dataset, and statistical code	22
**Appendices**			
Informed consent materials	#32	Model consent form and other related documentation given to participants and authorised surrogates	Supplement
Biological specimens	#33	Plans for collection, laboratory evaluation, and storage of biological specimens for genetic or molecular analysis in the current trial and for future use in ancillary studies, if applicable	n/a

None The SPIRIT Explanation and Elaboration paper is distributed under the terms of the Creative Commons Attribution License CC-BY-NC. This checklist can be completed online using

For peer review only

https://www.goodreports.org/, a tool made by the EQUATOR Network in collaboration with Penelope.ai

For peer review only

South West London and
St George's Mental Health
NHS Trust

Acceptance and Commitment Therapy (ACT) for Functional Cognitive Disorder (FCD) - ACT4FCD

Chief Investigator: Dr Norman Poole

Participant Information Sheet

We'd like to invite you to take part in our research study. Before you decide, it is important that you understand why the research is being done and what it would involve for you. Please take time to read this information and discuss it with others if you wish. If there is anything that is not clear, or if you would like more information, please contact the research team (see details at the bottom of this leaflet).

What is the aim of this study?

People with Functional Cognitive Disorder (FCD) suffer from memory problems that negatively impact their everyday life and personal wellbeing. At present treatment options are limited in number and accessibility. Acceptance and Commitment Therapy (ACT) focuses on changing a person's relationship with their thoughts and feelings, using mindfulness and acceptance. ACT has been successfully used in the treatment of conditions similar to FCD, and we have tested ACT on FCD in one small study where it appeared to work well.

Now we need to try it with a larger number of patients from different clinics and different backgrounds to make sure it is acceptable and useful to all patients. When we introduce a new way of working it is important that we compare it against our usual way of working and measure the results to see which way is best. To do this we will be allocating half (50%) of all people who take part into the usual care treatment group and half (50%) into the usual care plus specialist ACT group.

To try and make sure the groups are the same to start with, each participant is put into a group by chance (randomly). This process is called randomisation and to ensure that it is fair, the group you will be allocated to will be decided by a computer programme in a clinical trials unit. None of the researchers or members of the care team will have any input into which group you will be allocated to. The findings will help us to further develop specialist 'ACT' support programmes. This is a feasibility study (a practice-run before doing a large-scale study). It will help us find out more about:

For peer review only - http://bmjopen.bmj.com/site/about/guidelines.xhtml

South West London and St George's Mental Health NHS Trust

a. whether people with functional cognitive disorder find this intervention helpful; and how we can improve it.
b. whether people who have functional cognitive disorder find this type of trial acceptable and whether they are willing to be randomly allocated to receive the specialist support we have developed, in addition to their usual care; or receive only the usual care that is currently available to them.
c. how best to train staff to deliver this specialist support.
d. how best to measure the costs of that support and how well it works.
e. whether people taking part can complete the questionnaires we plan to use, without difficulty

Once we have completed the study and gathered information about each of these points, we can then make changes to prepare for conducting a larger study. We need to ensure that research meets patient needs and are asking your help to do this. If you are interested in taking part, please read the rest of this information sheet.

Why have I been invited?
We are asking you to take part because you have an established diagnosis of functional cognitive disorder that resulted in using one of the outpatient clinics and you are aged 18 or over. Your consultant has helped us to identify who to ask. We are inviting a total of 48 participants like you to take part.

Do I have to take part?
No. It is up to you to decide whether or not to take part. If you do decide to take part you will be

given this information sheet to keep and be asked to sign a consent form. If you decide to take part you are still free to withdraw at any time and without giving a reason. This would not affect your legal rights.

What will happen to me if I take part?
After you have consented, the research assistant will conduct an initial assessment to confirm you are eligible to take part in the study. If found to be eligible, you will be asked to complete several questionnaires assessing your quality of life, mood, memory, and how well you are functioning with the condition.

After this initial assessment the trial manager will contact King's College clinical trials unit (an external provider) who will allocate (randomise) you into one of the two groups, either the ACT intervention or standard medical care. The trial manager will then contact you to let you know which group you have been allocated to.

For peer review only - http://bmjopen.bmj.com/site/about/guidelines.xhtml

South West London and
St George's Mental Health
NHS Trust

Whichever arm of the trial you are allocated to, you will be asked to complete questionnaires on 4 separate occasions; at baseline, and again 2 months, 4 months and finally at 6 months after first being randomised. After completing them on this 4th and final occasion you will leave the study.

The questionnaires allow us to study whether the ACT intervention has been helpful and whether any improvements are maintained beyond the end of the intervention. A member of the research team will be on hand to assist with completing the questionnaires, should this be required. Each set of questionnaires will take between 35 - 45 minutes to complete.

What are the interventions I could receive?

You will receive either:

a. the usual care and access to services provided for people following a diagnosis of functional cognitive disorder within this Trust (consisting of the standard care already provided to you in the local memory clinics, neuropsychiatry service, or cognitive neurology clinic (wherever you were seen for assessment and diagnosis).
b. the usual care and access to services provided for people following functional cognitive disorder within this Trust **plus** the specialist ACT intervention being tested in this study.

There is a possibility that you may be disappointed by which group you have been allocated to, but each of the groups **is equally important** to developing this specialist ACT support programme and we hope that whatever the outcome you will continue to take part.

<u>During the 6 months in the study</u>

If you are allocated to the usual care group you will receive all the usual support and access to services provided by your care team.

If you are allocated to receive the specialist ACT programme from the clinician, you will be contacted by the treating clinician to arrange for the group intervention which consists of 4, 2-hour weekly treatment sessions, followed by 1, 2-hour booster session a month after the final treatment session. The ACT Group Intervention will host roughly 5-8 participants per group and will be entirely online. You will be expected to attend all 5 sessions if allocated to this group. Over the study period we will run groups on different days of the week and times of the day to maximise the chance of there being a group convenient to you. However, once

For peer review only - http://bmjopen.bmj.com/site/about/guidelines.xhtml

South West London and St George's Mental Health NHS Trust

allocated a particular group you will remain in that one for the duration of your intervention. They are run online to minimise disruption to your schedule.

The ACT intervention includes education about normal memory and forgetting. The intervention aims to decrease the threat of memory failures and to alter a person's relationship to their symptoms while encouraging behaviour that is in line with their values and goals. The concepts of "primary suffering" (the unwanted memory symptoms) and "secondary suffering" (attempts to control memory failures) are discussed. Brief mindfulness practices are incorporated into the sessions to improve acceptance, aiming for less distressing interpretations of unwanted experiences. Value-based goals are identified throughout the sessions to shift away from avoidance-based behaviour and the focus on memory symptoms.

What happens during a session?

All the sessions are run by two of the researchers who have expertise in delivering online group psychological therapies. In each of the sessions, specific topics are covered, and exercises undertaken. Each session begins with an overview of what will be covered and a reminder of what had been discussed at the previous one. Members of the group are encouraged to participate openly in these sessions and share their thoughts and experiences. Homework is set at the end of each session and discussed at the subsequent one. At the end of each session some tasks are given for trying out before the next session. Feedback is then shared at the next session. Participants are actively encouraged to engage in sessions as this appears to strengthen positive outcomes of the treatment. Everything discussed is confidential so must not be shared outside the group, and this will be explained again at the start of each session.

Recording of Sessions

All sessions of the ACT Intervention will be video recorded using MS Teams. The purpose of recording is not to review participants, but to ensure that the ACT process has been followed. The recordings will be reviewed for ACT fidelity and deleted at the end of the trial. Participants have the right to switch off their camera during sessions if they do not wish to be recorded.

Follow-up

While in the study (regardless of which group you are in) we would like to follow your progress and will ask you to complete questionnaires (either online or paper copies can be sent to you) at the **start** of the study and again at **2**, **4** and **6** months. When these questionnaires are due the research assistant will contact you to confirm that you are happy to continue and arrange a mutually convenient time to meet with you in person or online to support you in completing

For peer review only - http://bmjopen.bmj.com/site/about/guidelines.xhtml

South West London and St George's Mental Health NHS Trust

the questionnaires. These questionnaires assess how you are doing so are very important for the study.

We will also collect information from you and your medical notes about the amount of support you have received previously and which services you have accessed. We will only collect information directly relevant to your participation in this study and nothing else.

Additionally, as part of a sub-study, we may wish to interview you for about 45-60 minutes face-to-face, either via an online videocall or in person. This interview will be audio recorded and then transcribed. Like all of your data, the recording and transcription will be anonymised. We will ask you about the support you have received and the things you found useful or most helped you in your day-to-day functions. If you are invited to attend the interview, this will be a one-off occurrence and entirely voluntary, and you will be given further information beforehand so that you can decide whether you would like to be interviewed or not. **You do not have to agree to this interview to be able to take part in the study.**

Travel expenses and payment for participation

Participants will receive £25 for taking part in the trial, plus reimbursement of any travel costs involved, and £10 for completing each full set of questionnaires. We will also pay £20 to those who take part in the additional 45-60 minutes interview.

What are the possible disadvantages and risks of taking part?

We do not expect there are any disadvantages or risks to you. We will arrange any interviews at times to suit you. You may feel anxious before or tired after taking part in the sessions or while completing the questionnaires, but we will do everything we can to minimise or prevent this. You will be asked about your well-being over the course of the study, and you will have the opportunity to take short rest breaks when completing the questionnaires.

You may find the ACT sessions tiring or inconvenient, however, the timing of these will be arranged to be maximally convenient to participants. We will run groups on two days of the week at different times so if one group runs at a time that is inconvenient then you will have the option of an alternative day/time.

What are the possible benefits of taking part?

We cannot promise that being in either arm of this trial will help you but the information we obtain will help us plan a larger study to test how effective the ACT intervention is. In the future, this could help improve services for other people who have a functional cognitive disorder.

For peer review only - http://bmjopen.bmj.com/site/about/guidelines.xhtml

South West London and St George's Mental Health NHS Trust

What happens when the study stops?

When the study ends after 6 months, you will continue with your usual care from your hospital or GP.

What will happen if I don't want to carry on with the study?

Your participation is voluntary, and you are free to withdraw at any time, without giving any reason, and without your legal rights being affected. If you withdraw then the information collected so far cannot be erased and this information may still be used in the project analysis.

What if there is a problem?

If you have a concern about any aspect of this study, you should ask to speak to the Principal Investigator Dr Norman Poole or Trial Manager Dr Serena Vanzan (contact details below) who will do their best to answer your questions.

If you remain unhappy and wish to complain formally, you can do this through the NHS Complaints Procedure. Details can be obtained from the Trust's Patient Advice and Liaison Service (PALS). Tel: 0203 513 6150 (Monday - Friday 9.30am to 4.30pm) or email pals@swlstg.nhs.uk.

How will we use information about you?

We will need to use information from your medical records for this research project. This information will include your:

- NHS/Hospital number
- Name
- Contact details
- Ethnicity
- Gender
- DoB
- Relationship status
- Employment status
- Current medication
- Medical history

People will use this information to do the research or to check your records to make sure that the research is being done correctly. People who do not need to know who you are will not be able to see your name or contact details. Your data will have a code number instead. We will keep all information about you safe and secure.

Once we have finished the study, we will keep some of the data so we can check the results. We will write our reports in a way that no-one can work out that you took part in the study.

For peer review only - http://bmjopen.bmj.com/site/about/guidelines.xhtml

South West London and St George's Mental Health NHS Trust

What are your choices about how your information is used?

You can stop being part of the study at any time, without giving a reason, but we will keep information about you that we already have.

- We need to manage your records in specific ways for the research to be reliable. This means that we won't be able to let you see or change the data we hold about you.
- If you agree to take part in this study, you will have the option to take part in future research using your data saved from this study. If you consent to this we may:
  - Use your data already collected for this study in future research.
  - Contact you regarding taking part in future research relating to this current study.

Where can you find out more about how your information is used?

You can find out more about how we use your information

- at www.hra.nhs.uk/information-about-patients/
- by asking one of the research team
- by sending an email to SWLSTG's Data Protection Officer, John Hughes (john.hughes@swlstg.nhs.uk)
- by ringing us on 020 8725 3786

Will my taking part in this study be kept confidential?

We will follow ethical and legal practice and all information about you will be handled in confidence.

If you join the study, some parts of your medical records and the data collected for the study will be looked at by authorised persons from South West London Mental Health Trust, who are organising the research, and the King's Clinical Trials Unit. It may also be looked at by authorised people to check that the study is being carried out correctly. All will have a duty of confidentiality to you as a research participant and we will do our best to meet this duty.

All information which is collected about you during the course of the research will be kept strictly confidential, stored in a secure and locked office, and on a password protected database. Any information about you which leaves the hospital will have your name and address removed (anonymised) and a unique code will be used so that you cannot be recognised from it.

Your personal data (address, telephone number) will be kept for 6 months after the end of the study so that we are able to contact you about the findings of the study and possible follow-up studies (unless you advise us that you do not wish to be contacted). All other data

For peer review only - http://bmjopen.bmj.com/site/about/guidelines.xhtml

South West London and St George's Mental Health NHS Trust

(research data) will likewise be kept securely for 5 years after completion of the whole study (not just your involvement in it). After this time your data will be disposed of securely. During this time all precautions will be taken by all those involved to maintain your confidentiality, only members of the research team will have access to your personal data.

Although the information we collect about you is confidential, should you disclose anything to us which we feel puts you or anyone else at risk, we may feel it necessary to report this to the appropriate persons.
We would ask for your permission for the anonymised data set to be used to inform future projects and for education purposes and this permission is included within your consent form.

Involvement of the General Practitioner (GP)
If you do decide to take part in the study, we will inform your GP and provide them with a copy of this information sheet.

What will happen to the results of the research study?
We will use these findings to support the design of a large-scale study to test whether this ACT intervention results in people with functional cognitive disorder having better quality lives. The findings will be written up and submitted for publication to enable other NHS services to learn from our experiences. All reports and publications will be anonymised, and you would not be identified in any report or publication.

Who is organising and funding the research?
The study is being organised by the South West London and St Georges Mental Health NHS Foundation Trust (SWLSTG) and is being funded by National Institute for Health and Care Research (NIHR) Programme (project number NIHR202743).

Who has reviewed the study?
All research in the NHS is looked at by independent groups of people, called a Research Ethics Committee to protect your interests. This study has been reviewed and received a favourable ethical opinion from South East Scotland REC 02 (22-SS-0059).

Further information and contact details
If you have any questions about the study, wish to discuss taking part or have any concerns, you can contact the researchers leading the study:

Chief Investigator	Dr Norman Poole	act4fcd@swlstg.nhs.uk	07519668140
Trial Manager	Dr Serena Vanzan		
Research Assistant	Aimee Duffus		

For peer review only - http://bmjopen.bmj.com/site/about/guidelines.xhtml

South West London and St George's Mental Health NHS Trust

General information about taking part in research studies can also be obtained from your hospital's Patient Advice and Liaison Service (PALS), Tel: 0203 513 6150 (Monday - Friday 9.30am to 4.30pm) or email pals@swlstg.nhs.uk.

Many thanks for reading this information sheet. Please keep this information sheet. We will ask you to sign a consent form if you agree to take part and we will give you a copy of it to keep.

For peer review only

For peer review only - http://bmjopen.bmj.com/site/about/guidelines.xhtml

South West London and St George's Mental Health NHS Trust

Acceptance and Commitment Therapy (ACT) for Functional Cognitive Disorder (FCD)- ACT4FCD

Chief Investigator: Dr Norman Poole

Informed Consent Form

Participant Number:____________________

Please initial box

1. I confirm that I have read the information sheet dated 16.11.22 (version 2.1) for this study. I have had the opportunity to consider the information, ask questions and have had these answered satisfactorily. ☐
2. I understand that my participation is voluntary and that I am free to withdraw at any time without giving any reason, without my medical care or legal rights being affected. ☐
3. I understand that the data collected up to the point of my withdrawal will still be used for analysis. ☐
4. If randomly allocated to the ACT intervention group, I consent to be video recorded during therapy session. ☐
5. I understand that relevant sections of my medical notes and data collected during the study, may be looked at by individuals from the research team, where it is relevant to my taking part in this research. I give permission for these individuals to have access to my records. ☐
6. I understand my personal data will be kept for a maximum of 6 months following the end of the study and anonymised data will be made available to the research team to support future research. ☐
7. I agree to my General Practitioner being informed of my participation in the study. ☐

For peer review only - http://bmjopen.bmj.com/site/about/guidelines.xhtml

South West London and St George's Mental Health NHS Trust

8. I agree to take part in this study. ☐

9. I agree to be contacted to take part in an additional interview with the research team to share my experiences of living with FCD and of taking part in this trial. I understand that agreeing to be contacted does not oblige me to participate in the additional interview. Yes ☐ No ☐

10. I agree to be contacted to take part in future research regarding treatment for FCD. I understand that agreeing to be contacted does not oblige me to participate in any further studies. Yes ☐ No ☐

11. I would like to receive the study outcomes at the end of the study. Yes ☐ No ☐

**Name of Participant**	**Date**	**Signature**
Name of Person taking consent	Date	Signature

For peer review only

For peer review only - http://bmjopen.bmj.com/site/about/guidelines.xhtml

Summary of ACT for FCD Group Intervention

The group includes psychoeducation on normal memory functioning, in particular the role of attention and normal patterns of forgetting. This psychoeducation element aims to reduce the threat of cognitive symptoms. In line with the ACT model, the group aim is to increase psychological flexibility in response to the symptoms and associated thoughts and feelings. ACT is a contextual cognitive-behavioural therapy approach. Instead of targeting specific thoughts in relation to unwanted symptoms, it focuses on altering a person's relationship with their symptoms, increasing acceptance of the symptoms, and encouraging behaviour that is in line with a person's values (moving away from behaviour that focuses on avoidance of symptoms).

The concepts of "primary suffering" (the unwanted cognitive symptoms) and "secondary suffering" (attempts to control cognitive "failures" causes additional suffering, such as ruminations, negative predictions, and avoidance) are introduced. It is explicitly stated that the intervention does not aim to reduce "primary suffering" (cognitive symptoms), although it is possible that improvements may occur if secondary suffering is reduced. Brief mindfulness practices are incorporated in the group to facilitate "bottom up" processing, acceptance, and more neutral interpretations of unwanted experiences. Value-based goals are identified throughout the sessions to shift away from avoidance-based behaviour and the focus on cognitive symptoms.

The intervention protocol will be amended in light of specific feedback received during the contemporaneous qualitative interviews which are designed to explore the participants experience of and satisfaction with the groups.

Summary of the session content

	Group content
**Session 1**	Ground rules/house-keeping<br>Introductions<br>Psychoeducation (models of memory, normal forgetting, attention, functional cognitive disorder, fight/flight response)<br>Primary and secondary suffering<br>Vicious cycles in FCD<br>Brief mindfulness practice<br><br>Home practice:<br>Read Session 1 handout and write down any questions
**Session 2**	Brief mindfulness practice

For peer review only

	Check-in regarding home practice<br>What is ACT<br>Mindfulness<br>Values<br><br>Home-practice:<br>Mindfulness practice<br>Read Session 2 handout and write down any questions
**Session 3**	Brief mindfulness practice<br>Check-in regarding home practice<br>Value-based action<br>Value-based goals (passengers on the bus metaphor)<br><br>Home practice:<br>Working towards value-based goal<br>Mindfulness practice<br>Read Session 3 handout and write down any questions
**Session 4**	Brief mindfulness practice<br>Check-in regarding home practice<br>Cognitive fusion: Thoughts as barriers<br>Complete Willingness & Action plan<br><br>Home practice:<br>Working towards value-based goal<br>Mindfulness practice<br>Read Session 4 handout and write down any questions
**Session 5 (booster)**	Review goals<br>Trouble-shooting

Summary of ACT for FCD Group Intervention

The group includes psychoeducation on normal memory functioning, in particular the role of attention and normal patterns of forgetting. This psychoeducation element aims to reduce the threat of cognitive symptoms. In line with the ACT model, the group aim is to increase psychological flexibility in response to the symptoms and associated thoughts and feelings. ACT is a contextual cognitive-behavioural therapy approach. Instead of targeting specific thoughts in relation to unwanted symptoms, it focuses on altering a person’s relationship with their symptoms, increasing acceptance of the symptoms, and encouraging behaviour that is in line with a person’s values (moving away from behaviour that focuses on avoidance of symptoms).

The concepts of “primary suffering” (the unwanted cognitive symptoms) and “secondary suffering” (attempts to control cognitive “failures” causes additional suffering, such as ruminations, negative predictions, and avoidance) are introduced.

For peer review only - http://bmjopen.bmj.com/site/about/guidelines.xhtml

It is explicitly stated that the intervention does not aim to reduce “primary suffering” (cognitive symptoms), although it is possible that improvements may occur if secondary suffering is reduced. Brief mindfulness practices are incorporated in the group to facilitate “bottom up” processing, acceptance, and more neutral interpretations of unwanted experiences. Value-based goals are identified throughout the sessions to shift away from avoidance-based behaviour and the focus on cognitive symptoms.

The intervention protocol will be amended in light of specific feedback received during the contemporaneous qualitative interviews which are designed to explore the participants experience of and satisfaction with the groups.

For peer review only

South West London and St George's Mental Health NHS Trust

Data Management Plan (DMP)

Acceptance and Commitment Therapy (ACT) for Functional Cognitive Disorder (FCD) - ACT4FCD

POON1002

1. Trial information

Trial type	Non-CTIPM Intervention - Feasibility
Study Sites	Single-site (SWLSTG)
Total sample size	48
Total duration of study (months)	Trial start date _01/05/2022__<br>Planned duration for recruitment (months)___6____<br>Planned duration of follow-up (months)___6____<br>Total Duration ___24____Months
Study objective and design	We aim to study the feasibility of delivering a Randomised Control Trial (RCT) of an online group intervention based on Acceptance and Commitment Therapy (ACT) adapted for those with FCD. The treatment aims to reduce the threat of the memory failures and improve quality of life despite their presence. Participants will be recruited via SWLSTG and St George's neuropsychiatry services and memory clinics. All those eligible will be randomly allocated to either 5 sessions of online group ACT for FCD or treatment as usual. Questionnaires measuring various dimensions of physical, cognitive and mental wellbeing will be administered 2, 4 and 6 months after initial baseline. We will compare the outcome of those who receive the intervention with treatment as usual.

For peer review only - http://bmjopen.bmj.com/site/about/guidelines.xhtml

South West London and St George's Mental Health NHS Trust

	A selection of participants will be invited to take part in interviews about their lived experiences of FCD as well as to receive feedback on the study intervention.
Primary outcome measure	Increased psychological flexibility
Secondary outcome measures	Self-evaluations (questionnaires) of:<br>• Memory<br>• Anxiety<br>• Depression<br>• Global functioning<br>• Quality of life<br>• General wellbeing<br>• Service utilisation
Interim analysis	Yes/**no**, if yes when_____________(Months from baseline)

2. Trial personnel and contact details

This section details the name, their position in the trial, email address, telephone/fax number for all staff involved in the trial including the sponsor. The trial coordinator/trial manager, the investigators, study staff involved in the data management (including computing staff responsibilities for maintaining hardware and software), the monitors and anyone else associated with the trial at each site.

2.1 Sponsor site personnel (add or remove accordingly)

Role	Name	Organisation	Contact Details
Sponsor	SWLSTG R&D	SWLSTG	ResearchDevelopment@swlstg.nhs.uk
Chief Investigator	Norman Poole	SWLSTG	Norman.Poole@swlstg.nhs.uk
Trial Manager	Serena Vanzan	SWLSTG	Serena.Vanzan@swlstg.nhs.uk
Research Assistant	Aimee Duffus	SWLSTG	Aimee.Duffus@swlstg.nhs.uk
Research Assistant	Rebecca Cox	SWLSTG	Rebecca.Cox@swlstg.nhs.uk
Research Assistant	Tasnim Fakira	SWLSTG	Tasnim.Fakira@swlstg.nhs.uk
Co-Investigator and Trial statistician	Jared Smith	SGUL	jasmith@sgul.ac.uk
Co-Investigator and Trial statistician	Nadia Mantovani	SGUL	nmantova@sgul.ac.uk
Co-Investigator	Sarah Cope	SWLSTG	Sarah.Cope@swlstg.nhs.uk

For peer review only - http://bmjopen.bmj.com/site/about/guidelines.xhtml

Co-Investigator	Mark Edwards	KCL	mark.j.edwards@kcl.ac.uk
Trial statistician	Barbara Barrett	KCL	barbara.m.barrett@kcl.ac.uk
Primary contact for DM issues	Serena Vanzan	SWLSTG	Serena.Vanzan@swlstg.nhs.uk
Secondary contact for DM issues	Norman Poole	SWLSTG	Norman.Poole@swlstg.nhs.uk

For peer review only

South West London and St George's Mental Health NHS Trust

3. Milestones

3.1 Study Milestones

Milestone	Date/Estimated Date
Date funding confirmed	23.07.2021
Date and version number of approved protocol	14.07.2022, v1.0
Date and version number of final protocol amendment(s)	n/a
Date/version number of final approved CRF	No approval required
Release date/version number of final database	Est end Oct 2022
Date DMP signed off	
Date of first participant first visit (FPFV)	Est 17.10.2022
Date last participant last visit (LPLV)	Est Sep 2023

3.2 Proposed Data Milestones

Milestone	Date/Estimated Date
Data entry to commence	n/a (data collected directly on database)
Date of interim data partial lock if applicable	n/a
Data entry completed	Est Sept 2023
Last query resolved in the system	Est Oct 2023
Date of database final lock	Est Oct 2023

4. Data collection & data entry system

4.1 Detail how data will be collected and entered from each site, whether to ***complete the paper CRFs*** *or how to enter data electronically from each site.*

CRFs and outcome measures (questionnaires) will be completed electronically via the software REDCap, either by the RA (CRFs) or the participant (questionnaires). Where the participant will prefer paper questionnaires, these will be provided and returned via post, and the RA will enter the responses onto REDCap.

In the event that the online database will not be finalised at the time of recruitment opening, paper CRF and Questionnaires will be completed by the RA and participants, and the data will be entered onto REDCap as soon as this will become available.

Interview data (audio) will be automatically transcribed into a Word file by Teams, and the files will be saved in the study folder on SWLSTG's shared drive.

4.2 Provide details on the system used for data entry

The REDCap online software will be used for collecting CRF and quantitative data.

4.3 Outline who will be conducting the data entry from each site?

The RAs and the participants themselves.

For peer review only - http://bmjopen.bmj.com/site/about/guidelines.xhtml

4.4 *Outline whether single or double data entry will be carried out for all sites*
Single.

4.5 *If double data entry is required describe the process and whether a 100% of CRFs or a sample of CRFs will be double entered. Outline how the two entries will be compared, who will carry out the comparison and how the results will be dealt with.*
N/a

4.6 *Outline the checks undertaken for outcome measures*
The TM will perform regular self-monitoring visits in which the data entered for a proportion of participants will be checked for completeness, accuracy and timely completion.

4.7 *Provide details on how the data will be centralised*
N/a – one site.

5. Data checks & data validation for each site

5.1 *Outline who will perform data cleaning, missing data checks, consistency checks, range checks and logic, and how these are checked at each site?*
The TM will perform regular self-monitoring visits in which the data entered for a proportion of participants will be checked for completeness, accuracy and timely completion. In addition, value range restrictions will be applied in REDCap so that no values outside the available range can be entered when completing closed questions.

5.2 *Describe how regularly the data will be checked*
Monthly.

5.3 *Provide contact person for each site for data queries if data managed centrally*
N/a

5.4 *Describe the data flow from each site to central data centre, and who will conduct the overall data check*
N/a – one site.

5.5 *Detail the **flow of the data** from the field to the final storage for each site*
Quantitative data will be collected directly on study database. Qualitative data (interviews) will be recorded on Teams (on a Trust laptop), transcribed by Teams and saved on the study's shared drive folder.

6. AE and SAE data handling

Outline how the AE data will be collected by each site and collated for all sites at the end of the trial.
N/a – one site.

7. Partial and/or final data check and database lock

For peer review only - http://bmjopen.bmj.com/site/about/guidelines.xhtml

South West London and St George's Mental Health NHS Trust

Detail if an interim analysis is planned in the trial protocol, stating the time point and whether the database will be partially locked for the interim analysis.
N/a

Detail the process for partial and final data checks and data lock, outline who checks the data and who will sign off for partial/final data lock form(s)?
The database will be locked after the following actions have been confirmed as completed by the TM:

1. All CRF data has been collected and entered onto REDCap.
2. All queries identified during regular data checks and self-monitoring visits have been resolved/clarified.
3. All missing information has been confirmed as being not available (as opposed to not entered).
4. All monitoring visits (incl. close-out) have been performed, and outstanding actions completed.
5. A final data quality check has been performed.

The TM will be responsible for locking the database (notifying the CI and Sponsor), send the relevant data to the trial statisticians (with treatment allocation coded to prevent unblinding, where relevant), and save a copy of the full database in the TMF.

8. Data security and transmission between sites

Provide details on the data security procedures for transmitting data between sites
To comply with SWLSTG's Information Governance regulations, the datasets will be sent via encrypted email to the trial statisticians.

9. Data export & analysis

Explain how data will be exported and who the data should be sent to for data analysis.
The qualitative data will be saved on Word files, sent via an encrypted email to Nadia Mantovani, Trial Statistician and Co-Investigator. The full database with demographic and questionnaire data will be exported in CSV format and sent via encrypted email to Jared Smith, Trial Statistician and Co-Investigator. The responses to the AD-SUS questionnaire will be exported in Excel format and sent via encrypted email to Barbara Barrett, Trial Statistician.

10. Data Back-up and archiving

Describe procedures in place to ensure data protection including back-up system (if you don't do this you could lose the data!)
The database (all data on REDCap) will be downloaded and backed up weekly on a Trust external hard drive. The TMF, including the interview transcriptions, will be saved on the Trust's shared drive, which is backed up every night, in a restricted folder dedicated to the study. In addition, the TMF will also be backed up once a week on the Trust encrypted external hard drive.

For peer review only - http://bmjopen.bmj.com/site/about/guidelines.xhtml

BMJ Open

Feasibility randomised controlled trial of online group Acceptance and Commitment Therapy for Functional Cognitive Disorder (ACT4FCD)

Journal:	*BMJ Open*
Manuscript ID	bmjopen-2023-072366.R1
Article Type:	Protocol
Date Submitted by the Author:	04-Apr-2023
Complete List of Authors:	Poole, Norman; South West London and Saint George's Mental Health NHS Trust,<br>Cope, Sarah; South West London and Saint George's Mental Health NHS Trust<br>Vanzan, Serena; South West London and Saint George's Mental Health NHS Trust<br>Duffus, Aimee; South West London and Saint George's Mental Health NHS Trust<br>Mantovani, Nadia; St George's Hospital<br>Smith, Jared; St George's Hospital<br>Barrett, Barbara; King's College London<br>Tokley, Melanie; Eastern Community Mental Health Centre<br>Scicluna, Martin; South West London and Saint George's Mental Health NHS Trust<br>Beardmore, Sarah; South West London and Saint George's Mental Health NHS Trust<br>Turner, Kati; St George's University of London<br>Edwards, Mark; King's College London Institute of Psychiatry Psychology and Neuroscience<br>Howard, Robert; University College London Division of Psychiatry
**Primary Subject Heading**:	Mental health
Secondary Subject Heading:	Neurology
Keywords:	PSYCHIATRY, NEUROLOGY, Delirium & cognitive disorders < PSYCHIATRY

SCHOLARONE™
Manuscripts

Feasibility randomised controlled trial of online group Acceptance and Commitment Therapy for Functional Cognitive Disorder (ACT4FCD)

Authors

Norman Poole,* Sarah Cope, Serena Vanzan, Aimee Duffus, Nadia Mantovani, Jared Smith, Barbara Barrett, Melanie Tokley, Martin Scicluna, Sarah Beardmore, Kati Turner, Mark Edwards, Rob Howard

Affiliations

NP: norman.poole@gmail.com (*corresponding author) South West London & St George's Mental Health NHS Trust; St George's Hospital Medical School (London, UK)

SC: sarah.cope@swlstg.nhs.uk South West London & St George's Mental Health NHS Trust (London, UK)

SV: serena.vanzan@swlstg.nhs.uk South West London & St George's Mental Health NHS Trust (London, UK)

AD: aimee.duffus@swlstg.nhs.uk South West London & St George's Mental Health NHS Trust (London, UK)

NM: nmantova@sgul.ac.uk South West London & St George's Mental Health NHS Trust; St George's Hospital Medical School (London, UK)

JS: jasmith@sgul.ac.uk South West London & St George's Mental Health NHS Trust; St George's Hospital Medical School (London, UK)

BB: barbara.m.barrett@kcl.ac.uk King's Health Economics, King's College London (London, UK)

MT: melanie.tokley@sa.gov.au Eastern Community Mental Health Centre (Adelaide, Australia); Rose Park Psychology (Adelaide, Australia)

For peer review only

MS: scicluna.mg@gmail.com PPI Rep, South West London & St George's Mental Health NHS Trust (London, UK)

SB: s_beardmore@hotmail.co.uk PPI Rep, South West London & St George's Mental Health NHS Trust (London, UK)

KT: creusk@aol.com South West London & St George's Mental Health NHS Trust; St George's Hospital Medical School (London, UK)

ME: mark.j.edwards@kcl.ac.uk Institute of Psychiatry, Psychology and Neuroscience, King's College London; South London and the Maudsley Hospital (London, UK)

RH: robert.howard@ucl.ac.uk University College London Division of Psychiatry (London, UK)

Word count

4021

Protocol version number & date

Version 2.0, 14/11/2022

For peer review only

Article Summary

Abstract

Introduction

Functional cognitive disorder is seen increasingly in clinics commissioned to assess cognitive disorders. Patients complain of frequent cognitive, especially memory, failures. The diagnosis can be made clinically, and unnecessary investigations avoided. While there is some evidence that psychological treatments can be helpful, they are not routinely available. Therefore, we have developed a brief psychological intervention using the principles of Acceptance and Commitment Therapy (ACT) that can be delivered in groups and online. We are conducting a feasibility study to assess whether the intervention can be delivered within a randomised controlled trial. We aim to study the feasibility of recruitment, willingness to be randomised to intervention or control condition, adherence to the intervention, completion of outcome measures, and acceptability of treatment.

Methods and analysis

We aim to recruit 48 participants randomised 50:50 to either the ACT intervention and Treatment as Usual (TAU), or TAU alone. ACT will be provided to participants in the treatment arm following completion of baseline outcome measures. Completion of these outcome measures will be repeated at 8, 16, and 26 weeks. The measures will assess several domains including psychological flexibility, subjective cognitive symptoms, mood and anxiety, health related quality of life and functioning, healthcare utilisation, and satisfaction with care and participant-rated improvement. Fifteen participants will be selected for in-depth qualitative interviews about their experiences of living with FCD and of the ACT intervention.

Ethics and dissemination

The study received a favourable opinion from the South East Scotland Research Ethics Committee 02 on 30th September 2022 (REC reference: 22/SS/0059). HRA approval was received on 1st November 2022 (IRAS 313730). The study has been registered with the ISRCTN (ISRCTN12939037). The results will be published in full in an open access journal.

ISRCTN Registration

https://doi.org/10.1186/ISRCTN12939037

Grant Number

National Institute for Health and Care Research (NIHR) grant number: NIHR202743

Strengths and limitations of this study

- Novel intervention with potential to be delivered at scale
- Adherence to principles of an evidence-based intervention (ACT)
- Utilising qualitative and quantitative methodologies which is key to contextualising patient experiences in a clinically meaningful measurement framework
- Recruiting from the full range of services that assess cognition in adults in the UK
- Participants not blinded to their intervention

Keywords

Functional Neurological Disorder

Cognitive Symptoms

Acceptance and Commitment Therapy

Randomized Controlled Trial

For peer review only

Introduction

Functional Cognitive Disorder (FCD) is defined as a complaint about memory function or other cognitive process in the absence of relevant neuropathology and with evidence of inconsistency between symptoms reported, objective signs, and known phenomenology of dementia syndromes.[1] Typical complaints include forgetting an intended action while in the process of carrying it out; inability to recall well-founded memories (such as PIN numbers and names); disruptions in the flow of thoughts and conversations; word-finding difficulties; and spoonerisms. [2] While anxiety and depression are common co-morbidities, they are generally mild and do not account for the severity or nature of the symptoms reported. Patients who experience FCD tend to perform as well or a little worse on neuropsychological tests as healthy controls but better than those with mild cognitive impairment and early Alzheimer's disease.[3] Disturbance of attention is thought to be responsible for the symptoms,[4] as with other functional neurological disorders,[5] although the underlying pathophysiological mechanisms remain unknown.

Diagnostic Memory Clinics (DMCs) are funded by clinical commissioning groups and run either by local mental health services or in partnership with acute trusts. Cognitive Neurology Clinics (CNCs) are increasingly provided by acute trusts and target younger populations with potential young onset dementias, and are more likely to see patients with FCD. As access to these services has improved, an increasing proportion of attendees are being diagnosed with FCD.[6] To date, the population prevalence of FCD has not been studied. However, a recent systematic review described 56 studies which demonstrated a high prevalence of cognitive symptoms in community populations (30% in 245,654 subjects).[3] The same review reported that 24% of DMCs attendees may have FCD, or one of its synonyms (range: 12–56%).

Those with FCD seeking referral to memory clinics have elevated levels of distress, depression, and anxiety.[3] Their symptoms persist and adversely impact employment status and activities of daily living (ADLs). Schmidtke and colleagues [7] found that cognitive symptoms persisted in 85% of those followed-up for an average of 20 months. One of their cohort (2.1%) went on to develop dementia, in keeping with the rate of revision of functional diagnoses and

For peer review only

neurological conditions generally.[8] Hence, FCD should not be regarded as the precursor for an inevitable dementia.

It is unclear how to help this group of patients. A recent survey of DMCs found 73% immediately discharged them to primary care and treatments offered ranged from simple reassurance to referral to a community mental health team.[9] As they receive little explanation for their symptoms, patients are liable to present to their GP requesting further referrals and investigations, with the potential for iatrogenic harm and unnecessary healthcare costs.

There is now preliminary evidence that strategies focused on expectations, cognitive restructuring and psychoeducation can be helpful.[10, 11] A recent meta-analysis of treatment studies to date found that group interventions involving both cognitive-training and expectancy-modification significantly improved psychological well-being. While expectancy-change interventions had little effect on objective measures of cognitive functioning, cognitive-training was associated with small, clinically insignificant improvements in tasks related to the training ones, with no generalisation to daily life.[11] No adverse events were described.

A rare, high quality RCT included in the meta-analysis involved 18 patients receiving 13 sessions of group cognitive-behavioural therapy (CBT) intended to change participants' beliefs and expectations.[10] Patients in the treatment group had significantly better memory-related self-efficacy than the controls at the end of the intervention and at 6-month follow-up (n=18). However, such interventions are not routinely provided by diagnostic memory, cognitive neurology, or neuropsychiatry clinics, where these patients are most likely to be seen. Furthermore, a 13-session intervention is resource intensive and unfeasible within DMCs and CNCs. Increasing Access to Psychological Therapies (IAPT) services are unlikely to offer any intervention beyond support for any co-morbid anxiety or depressive disorder.

St George’s Hospital in South London, in collaboration with South West London and St George’s NHS Mental Health Trust, is a centre of excellence for functional neurological disorders. We have developed a five-session group treatment based on the third-wave

For peer review only

cognitive behaviour therapy known as Acceptance and Commitment Therapy (ACT). This intervention focuses on changing a person's relationship with their thoughts and feelings. It makes use of mindfulness and acceptance processes and increases values-driven behaviour.[12] ACT considers psychological inflexibility – behaviour that is driven by attempts to excessively control internal experiences (such as difficult thoughts and feelings – as a source of emotional distress; hence, its aim is to enhance psychological flexibility.[13] Psychological flexibility is defined as "the ability to contact the present moment more fully as a conscious human being, and to change or persist in behaviour when doing so serves valued ends"[14] while psychological inflexibility is regarded a trans-diagnostic process common to numerous psychopathological states.[15]

Perceived threat is thought to be a maintenance factor for FND. Symptoms, such as cognitive failures in the case of FCD, are experienced as threatening, which causes hypervigilance and autonomic arousal. Symptoms are therefore experienced in a "top-down" manner - influenced by cognitive and neurobiological processes of expectations and predictions of illness.[5, 16] Improvement with ACT occurs through six key processes that can be grouped as either "mindfulness and acceptance processes" or "commitment and behaviour change processes".[12] Consequently, ACT aims to change a person's relationship with their internal experiences, increasing psychological flexibility and altering the top-down expectations by facilitating bottom-up processes, e.g., enhancing connection with direct experiences through mindfulness practices.

ACT's efficacy across a range of conditions has been demonstrated by several RCTs and meta-analyses. A recent review of 20 meta-analyses found it to be superior to inactive controls, treatment as usual (TAU), and active interventions (excluding CBT), with effect sizes ranging from small to medium.[17] There is evidence that ACT effectively reduces distress and disability in chronic pain [18] and long-term medical conditions [19] while it has also been recommended for the treatment of functional neurological disorders generally.[13, 20] ACT can be delivered in one-to-one or group formats. It is feasible and acceptable to deliver to patients with psychosis in a brief group format [21] and can be effectively delivered online through guided and unguided modules of learning.[22]

For peer review only

Unpublished initial pilot data suggested improvement in measures of quality of life and decreased psychological distress in four cohorts of patients who have received our brief ACT intervention. Following this, we were able to secure funding for a feasibility study from the National Institute for Health and Care Research (NIHR; grant number NIHR202743). We now aim to study the feasibility of delivering a randomised controlled trial of ACT for FCD as an online group intervention and compare this against current treatment as usual (TAU). We also aim to further refine the ACT intervention over the course of the study so it can be adapted and manualised for a future definitive randomised controlled trial.

Study objectives

The feasibility study aims to investigate:

- The willingness of clinicians in local services commissioned to assess patients with cognitive complaints to refer patients diagnosed with FCD into the study
- The willingness of patients with FCD to consent to the trial and be randomised to ACT+TAU vs TAU
- Acceptability of the online group ACT intervention
- Appropriateness and acceptability of clinical outcome measures
- Completion rates for outcome measures at the various time points and rate of adherence to the ACT intervention
- Fidelity of intervention
- Time needed to collect and analyse data
- Healthcare utilisation pre- and post-intervention
- Signal of efficacy in clinical outcomes

For peer review only

Methods

Trial design

ACT4FCD is a parallel-group, single-blind RCT, designed to assesses the feasibility of delivering a trial of a brief online group Acceptance and Commitment Therapy (ACT) and comparing it against the current standard intervention (Treatment As Usual (TAU)). Participants are assessed at baseline and again at 8 weeks, 16 weeks, and 26 weeks. In addition, the study aims to collect data on health utilisation before and after the intervention and has an embedded qualitative study of lived experience of FCD and the ACT intervention. This study adheres to the Standard Protocol Items Recommendations for Interventional Trials (SPIRIT; see Appendix 1).

Recruiting sites and participants

We are recruiting participants from Diagnostic Memory Clinics (DMCs), Neuropsychiatry Clinics (NPCs) Clinical Neurology Clinic (CNCs) in London. Recruitment began on 07/11/2022. These different clinics assess a spectrum of patients with cognitive symptoms. DMCs tend to see an older population while younger patients are more often referred to the NPC and CNC. If we are not successful recruiting participants from these sites alone then we will review within the trial management group and consider adding additional recruiting sites in nearby specialist clinics.

Clinicians in the recruiting clinics will refer potentially suitable patients to the study team and deliver the "treatment as usual" (TAU) intervention as part of routine clinical care. Potential participants will provide verbal consent to being contacted by the research team. Following this, they will receive the participant information sheet (see Appendix 2), including dates of the ACT groups, and the informed consent form (see Appendix 3) and given at least 24 hours to consider these. The consent form will also record whether participants are willing to be contacted about involvement in a parallel qualitative study, described below. Potential participants will be informed that choosing not to take part will not impact their medical treatment with any service. Once consent is given, the research assistant contacts the potential participant to complete a screening interview against the inclusion/ exclusion criteria (see Figure 1. for Study Flow Chart). The inclusion/ exclusion criteria are:

For peer review only

Inclusion criteria

- An established diagnosis of FCD made in DMC/CNC/NPC and confirmed by research team from review of clinical notes and examination findings
- Aged 18 or over
- Capacity to provide written informed consent

Exclusion criteria

- Disabling cognitive symptoms in the context of a primary psychiatric disorder (e.g. depression, severe generalised anxiety disorder, PTSD, bipolar affective disorder, schizophrenia).
- Greater than mild-moderate depressive or anxiety disorders (PHQ9 score ≥ 15 and/or GAD7 score ≥ 15)
- At "medium" or "high" risk of deliberate self-harm and/or suicide (based on clinical assessment)
- Another predominant functional disorder (e.g., functional seizures)*

*Co-morbid functional diagnosis is acceptable so long as those symptoms do not dominate the clinical picture.

For peer review only

- Diagnosis of dementia
- Diagnosis of learning disability
- Insufficient command of English to engage in conversation without an interpreter (as this would not be compatible with the online ACT intervention)

Primary outcome measures and progression criteria

The feasibility study is primarily gathering data on the feasibility of conducting a future definitive RCT. The primary outcome measures (and progression criteria) are:

- Rate of successful recruitment (≥70% intended participants recruited)
- Rate of successful adherence (≥75% in ACT + TAU attend four or more sessions)
- Acceptability of the ACT intervention (qualitative interview themes and majority (≥75%) satisfied/very satisfied on 5-point Likert scale)
- Signal of efficacy (based on increased psychological flexibility following intervention)

Randomisation

Participants will be randomised into ACT (plus TAU) or TAU using a simple block randomisation procedure (with randomly permuted block sizes of 2 and 4). Randomisation will be carried out via the online service Sealed Envelope by the Trial Manager, who will then inform participants of their arm allocation.

Blinding

Given the nature of the intervention, it is not possible to blind the participants to their intervention or those responsible for delivering the intervention (NP, SC, AD). The research assistant collecting the outcome data and the statistician will remain blind to treatment allocation (single-bind trial). Clinical outcome measures will be completed again at 8 weeks (T1), 16 weeks (T2), and 26 weeks (T3). Unblinding will be allowed only in case of an Serious Adverse Event (eg, resulting in death).

Secondary (clinical) outcome measures

Those who are deemed eligible to participate in the trial will be sent an individualised weblink to complete baseline (T0) clinical outcome measures. These can be completed on paper if the

For peer review only

participant prefers. The paper forms will be returned to the research assistant for inputting into the online database and then securely destroyed.

Clinical outcome measures will be completed at baseline (T0) and at 8 weeks (T1), 16 weeks (T2), and 26 weeks (T3). The proposed clinical outcome measures and the domains being measured include:

Health-related quality of life/functioning

- World Health Organisation Disability Assessment Schedule (WHODAS 2.0) [23]
- EuroQol 5-Dimension, 5-Level Health Scale (EQ-5D-5L) [24]
- ICEpop CAPability measure for Adults (ICECAP-A) [25]

Subjective cognitive symptoms

- Multifactorial Memory Questionnaire (MMQ) [26]

Depression and anxiety

- 9-question Patient Health Questionnaire (PHQ-9) [27]
- Generalized Anxiety Disorder 7 (GAD-7) [28]

ACT specific measure of change

- Acceptance and Action Questionnaire II (AAQ-II) [29] is a measure of psychological flexibility/inflexibility widely used in ACT. This would potentially be the primary outcome measure in a future definitive RCT.

Service utilisation and other cost variables

The Adult Service Use Schedule (AD-SUS) [30] is a self-report service use questionnaire completed by the study participant in interview with a trained researcher. The AD-SUS was developed by the study economist for previous work in similar populations and has been adapted for the needs of this study so that participants can complete this without assistance.

Improvement

- Clinical Global Impression – Improvement Scale (CGI-I) [31], single item, participant rated

For peer review only

Measure of satisfaction

- Satisfaction rating of treatment (single-item, 5-point Likert scale)

The various research activities and outcome measures and the time points when they are collected are listed in Table 1.

Table 1. Protocol schedule of procedures for the ACT4FCD study

	Screening	Baseline (T0)	2 months (T1)	4 Months (T2)	6 months (T3)
**Enrolment**					
Consent to contact obtained by clinical staff in DMC, CNC, or NPC	x				
Informed consent	x				
Contacted by RA to arrange screening interview	x				
**Screening**					
Diagnostic criteria for FCD	x				
Risk Assessment	x				
Demographics	x				
Co-morbidities	x				
Medications	x				
PHQ-9	x				
GAD-7	x				
Randomisation allocation		x			
**Interventions**		ACT+TAU	Intervention completed		
**Assessments**					
AAQ-II		x	x	x	X
MMQ		x	x	x	X
WHODAS 2.0		x	x	x	X
EQ-5F-5L		x	x	x	X
PHQ-9		x	x	x	X
GAD-7		x	x	x	X
AD-SUS		x			X
ICECAP-A		x	x	x	X
CGI-I					X

Likert 5-point satisfaction scale				x	
AEs/SAEs		x	x	x	X
**Qualitative Interviews**					
Informed consent for qualitative interview (select participants)		x	x		
Qualitative interviews with select sample of participants		x	x		

For peer review only

Participant payment

Participants will receive a non-contingent payment of £25 for taking part in the trial. To aid retention, participants will receive £10 at each time point clinical outcome measures are fully completed. Those who take part in the optional qualitative interviews (see below) will also receive an additional £20 incentive.

The ACT intervention

The group consists of psychoeducation about normal memory functioning, including the roles of attention and normal patterns of forgetting. The psychoeducation element aims to reduce the threat of cognitive symptoms. In line with the ACT model, the aim is to increase psychological flexibility (our proposed primary clinical outcome measure for a future RCT; Acceptance and Action Questionnaire II (AAQ-II)) in response to the symptoms and associated thoughts and feelings.

The concept of "secondary suffering" is introduced. [32] It is suggested that attempts to control cognitive "failures" leads to additional suffering, such as ruminations, negative predictions, and avoidance. It is explicitly stated that the intervention does not aim to reduce "primary suffering" (the cognitive symptoms), although it is possible that improvements *may* occur if secondary suffering is reduced. Brief mindfulness practices are incorporated in the

group to facilitate "bottom up" processing, acceptance, and more neutral interpretations of unwanted experiences. Value-based goals are identified throughout the sessions in order to shift away from avoidance-based behaviour and the focus on cognitive symptoms.

Participants will be given the time of all ACT sessions prior to randomisation and are asked to consent only if able to attend should they be randomised to the active intervention. They will sent an email with link and diary invite for all the sessions the week before the first session and follow up reminder calls/emails each week to maximise attendance and engagement despite their memory problems. If a participant fails to attend a session, they will receive a follow up call from the research assistant to ensure they had the time and link and to enquire about future attendance.

The intervention protocol (see Appendix 4) will be amended in response to specific feedback received during the contemporaneous qualitative interviews which are designed to explore the participants experience of and satisfaction with the groups.

The intervention group will also receive TAU, as below.

Treatment as usual

Treatment as usual (TAU) was selected as the fairest comparison, given that is what most patients in the UK currently receive within mental health and cognitive neurology settings. It consists of an explanation of the FCD diagnosis, provision of additional information about the diagnosis and underlying factors (such as medications, chronic pain, or poor sleep hygiene; information: https://www.neurosymptoms.org/en/symptoms/fnd-symptoms/functional-cognitive-symptoms), and signposting to local psychological services in primary care (Increasing Access to Psychological Therapies (IAPT)) for appropriate treatment, such as CBT, when co-morbid anxiety and/or depression have been identified. TAU will be delivered as part of routine clinical care by the recruiting service.

Withdrawal and non-adherence

Participants who do not attend out of the SF-GAPI modules or assessments are not replaced. Disclosed reasons for withdrawal, non-adherence or loss to follow-up are reported.

For peer review only

Sample size

This is a feasibility trial; as such, a power calculation is neither possible nor necessary. Rather, the sample size is pragmatic. Target recruitment is 48 patients in total (24 in each treatment arm), which provides sufficiently reliable estimates of feasibility outcomes such as recruitment, adherence and retention rates to inform a fully powered RCT. For example, assuming 70% of those approached consent to participate in the trial, the 95% confidence interval for adherence rate would have width of 13.0%. For those in the treatment arm, the 95% confidence interval for an intervention adherence rate of 80% would have width of 16.0%. A sample size of 48 is also consistent with those recommended for pilot and feasibility studies to provide adequate data and precision of means and variances (*n* between 24 and 50). [33, 34, 35] If it proves challenging to recruit participants via these routes alone then we will approach colleagues at other specialist services in London who see patients with FCD to increase our recruitment pool.

Statistical analysis plan

A fully documented statistical analysis plan, centred on describing key process measures to decide if a definitive trial is feasible, will be prepared by the statistician (SAP), formally agreed with co-investigators and the Trial Steering Committee prior to data collection being completed. Participant throughput will be summarized in an extended Consolidated Standards of Reporting Trials (CONSORT) diagram.[36] The CONSORT flow chart will be used to present descriptive data on trial referral, enrolment, intervention allocation, adherence, and retention and to document any deviations from protocol.

Feasibility outcomes will be summarised using descriptive statistics, with 95% confidence intervals (CIs) provided to permit assumptions when planning a future definitive trial. Data relating to referral, screening and enrolment, and recruitment logs will be used to produce accurate estimates of eligibility, consent, and recruitment rates.

Treatment assignation, intervention adherence (e.g., ACT session attendance) and satisfaction of care data will be used to contribute to the evaluation of the acceptability of randomisation and allocated intervention/treatment arms. At each time point, the time taken

For peer review only

(per participant) to complete trial measures will be recorded. Retention rates will also be estimated for each of the patient-reported/clinical outcome measures, with consideration given to differential dropout between the arms of the trial to identify potential (attrition) bias in treatment completion and/or data collection. All feasibility outcomes will be compared with full-trial progression criteria.

Baseline characteristics will be reported according to treatment arm. Continuous variables will be reported as mean (standard deviation (SD)) if normally distributed or median (inter-quartile range) if non-normal, while categorical variables will be presented as frequency (%). Subsequent analyses will summarise the proposed patient-reported and clinical outcomes (e.g., psychological flexibility, subjective memory function, quality of life, and depression/anxiety measures) at each time point for each trial arm using appropriate descriptive statistics (e.g., group mean, SD). To provide an indication of potential changes in scores/frequencies between the four time points, linear/logistic mixed effects regression models will be employed performed on an intention-to-treat (ITT) basis (accounting for data assumed to be missing at random). These random intercept (mixed) models will include intervention group, time, and intervention group by time interaction. There will be no emphasis on hypothesis testing, however, which is reserved for the future main trial. Rather, pre-to-post-intervention standardised effect sizes (Hedges' g, relative risk) will be computed (SDs will be computed from estimated model standard errors) with associated CIs calculated to explore imprecision around effect sizes.[37] Due to the small sample size, important covariates (e.g., baseline score on relevant measure, gender, age) may be included in models if the two arms happen to be highly imbalanced. ITT analyses will also be administered by imputing values for missing data using a conservative last observation carried forward (LOCF) procedure (given full sensitivity analysis testing of missing data assumptions is beyond the scope of a feasibility study). Exploratory analyses using mixed effect models will examine the rate of change in intervention and control groups on outcome measures across four time-points, adjusting for relevant baseline scores and variables of interest (anxiety, depression, subjective evaluation of memory), and investigate changes on a per-protocol basis (focused on intervention adherence; i.e., including only participants who attended at least 4 sessions and with post-treatment data).

For peer review only

The qualitative study

A sub-sample of participants in both arms of the trial will be invited for in-depth semi-structured interviews over the course of the intervention period. A sampling framework will be used that ensures participants are included that are representative of the sociodemographic characteristics and clinical profile. Interviews at baseline (T0) will focus on the experience of living with FCD and subsequent interviews (at T1), not necessarily with the same participants, will explore their views on the ACT intervention. These interviews will focus on the acceptability, number, and frequency of ACT sessions, ways of optimising engagement, perceived benefits/limitations of the intervention, and any recommendations for improvement of the components and/or content of the intervention.

Interview schedules will be coproduced by the PPI representatives and the research team. One-to-one, in-depth interviews will be carried out via Teams by the research assistant, recorded, and transcribed, and the transcripts cross-checked against the original recordings to ensure accuracy. The analysis will be led by the qualitative study expert and research assistant using reflexive thematic analysis [38] aided by NVivo12 software. The results of the T1 interviews will be utilised to optimise the intervention during the feasibility study with the aim of developing a formalised intervention to be used in a future RCT.

Data management

All data will be inputted by the participants themselves on to the trial database on REDCap and backed up weekly on a secure server. The electronic Trial Master File will be backed up weekly on an additional encrypted hard drive. No paper copies will be stored. Please refer to Data Management Plan (see Appendix 5) for details regarding confidentiality, data collection, data handling, and data transfer. The data collection and management will be in line with GDPR Data Protection Act (2018) and SWLSTG's Information Governance.

For peer review only

Patient and public involvement

The research design has been informed by our PPI representatives who were recruited from earlier pilots of the intervention conducted within the SWLSTG Neuropsychiatry Service. They assisted in the development of the intervention, study methodology, and review of clinical outcome measures. Two PPI participants (SB, MS) have been recruited to the Trial Steering Committee and are funded to assist with reviewing study materials, writing, and developing patient information leaflets, producing the semi-structured interview schedules for the qualitative study, and ensuring proper conduct of the study. We will document PPI activity over the course of the study in order to accurately assess where and how the lived experience perspective has been used and its impact on the research process and findings. All PPI representatives have lived experience of FCD and the proposed ACT intervention.

Serious adverse events

All adverse events (AEs) and serious adverse events (SAEs) reported spontaneously by participants or observed by researchers will be recorded and reported to the Trail Manager (TM) and Chief Investigator (CI). Urgent actions concerning participant and staff safety, communication with others, and clinical care will be immediately addressed by the CI and reported to the Trial Management Group (TMG). A summary of (S)AEs will be presented at each Trial Management Group (TMG) and Trial Steering Committee (TSC) meeting. AEs will be categorised for severity and seriousness by the TM and CI. SAEs will be further reviewed for relatedness to trial procedures and unexpectedness by the CI initially, and additionally by the chair of the TSC.

For peer review only

Ethics and dissemination

Ethics approval was sought from the South East Scotland Research Ethics Committee (REC) 02 and a favourable opinion was received on 30th September 2022 (REC reference: 22/SS/0059). Any amendments to the protocol will be agreed with the REC before being implemented and then amended on the ISCTRN Registry. The findings of the study will be published on an open access journal once the full trial has been completed. A data monitoring committee was not deemed necessary as participants are adverse effects are not expected in either of the randomisation groups.

For peer review only

Author contributions

NP, SC, and ME conceived the study. NP, SC, ME, NM, JS, and RH, developed and finalised the study design. JS provided statistical expertise in the clinical trial design and NM provided qualitative research expertise in the qualitative study design. BB contributed to the design of the health economics analysis. SB, MS, and KT provided expert by experience expertise. All authors contributed to refinement of the study protocol. NP drafted the manuscript. SC, SV, AD, NM, JS, BB, MT, MS, SB, KT, ME, and RH provided critical revisions to the manuscript and approved the final manuscript. NP is grant holder. NP, SC, SV, JS, ME, RH, and BB will have access to the final trial dataset. The authors intend to share deidentified individual clinical trial participant-level data after publication of results with researchers who provide a methodologically sound proposal. Requests can be sent to norman.poole@gmail.com

Study sponsor details

South West London & St George's Mental Health NHS Trust (SWLSTG)

Sponsor contact:
Tania West
R&D Governance & Clinical Trials Manager
South West London & St George's Mental Health NHS Trust
Research & Development, Newton Building (1st Floor)
Springfield University Hospital
Glenburnie Road
London SW17 7DJ
Email: researchdevelopment@swlstg.nhs.uk

For peer review only

Sponsor roles and responsibilities

SWLSTG Mental Health NHS Trust is not involved in any aspect of the study design and management, or in its data collection, analysis or interpretation. The Sponsor is responsible for providing the research team with adequate arrangements to successfully complete the trial, and to monitor its compliance with ethical guidelines and legislation. SWLSTG Mental Health NHS Trust is the data controller for the study and all data shall return to the Sponsor at the end of the trial. A collaboration agreement is being finalised with King's College London, where a Co-Investigator and a Trial Statistician are based. No Data Sharing Agreements are required.

Funding statement

This study is funded by the NIHR Research for Patient Benefit (RfPB) Programme (NIHR202743) and supported by the NIHR Clinical Research Network. The views expressed are those of the author(s) and not necessarily those of the NIHR or the Department of Health and Social Care.

Funder roles and responsibilities

NIHR is not involved in any aspect of the study design and management, or in its data collection, analysis or interpretation. Core publications (including, but not limited to, research protocol and results) were agreed with NIHR before the beginning of the trial, and NIHR will be informed of any additional publications. NIHR will also be acknowledged in any public dissemination of the research, including peer-reviewed articles, conference presentations and patient involvement activities.

Trial steering committee roles and responsibilities

The TSC will oversee the study on behalf of the of the trial Sponsor and Funder and ensure that the study is conducted within appropriate NHS and professional ethical guidelines. It will provide advice on all appropriate aspects of the project; will oversee progress of the trial, adherence to the protocol, participant safety and the consideration of new information of

For peer review only

relevance to the research question; will ensure the rights, safety and well-being of the participants are given the most important considerations; will ensure appropriate ethical and other approvals are obtained in line with the project plan; will agree proposals for substantial protocol amendments and provide advice to the Sponsor and Funder regarding approvals of such amendments. It will comprise the CI (NP), TM (SV), independent Chair (Professor Michael Kopelman), independent member (Dr Laure McWhirter), sponsor representative, and two independent PPI members.

Trial management group roles and responsibilities

The TMG is composed of AD, BB, JS, ME, NM, NP, RH, SB, SC, SV. The agreed terms of reference are:

1. To review and provide feedback on any amendment of the study protocol, outcome measures and recruitment documents
2. To support the progress of the trial towards its interim and overall objectives and against the study timescale
3. To agree on a trial monitoring plan
4. To advise on participant recruitment and retention
5. To facilitate data analysis
6. To help troubleshoot challenges to the timely completion of the trial
7. To review the study outcomes
8. To discuss future developments of the study
9. To report to the Trial Steering Committee (TSC)

Data availability statement

The data that support the findings of this study will be openly available in ISRCTN Registry at https://doi.org/10.1186/ISRCTN12939037

For peer review only

Competing interests statement

No competing interests

For peer review only

References

1. Ball HA, McWhirter L, Ballard C, et al. Functional cognitive disorder: dementia's blind spot. *Brain* 2020;143(10):2895-2903. doi.org/10.1093/brain/awaa224

2. Poole N, Cope S, Bailey C, Isaacs J. Functional cognitive disorders: Identification and management. *BJPsych Advances* 2019; **25(6)**: 342-350. doi:10.1192/bja.2019.38

3. McWhirter L, Ritchie C, Stone J, Carson A. Functional cognitive disorders - a systematic review. *Lancet Psychiatry*, 2020;**7(2)**: 191-207. doi: 10.1016/S2215-0366(19)30405-5

4. Teodoro T, Edwards M, Isaacs J. A unifying theory for cognitive abnormalities in functional neurological disorders, fibromyalgia and chronic fatigue syndrome: systematic review. *J Neurol Neurosurg Psychiatry* 2018;**89**:1-12. doi:10.1136/jnnp-2017-317823

5. Edwards MJ, Adams RA, Brown H, et al. A Bayesian account of 'hysteria'. *Brain* 2012;**135(11)**:495–512.

6. Larner AJ Impact of the National Dementia Strategy in a neurology-led memory clinic: 5-year data. *Clinical Medicine* 2014;**14**:216. doi: 10.7861/clinmedicine.14-2-216.

7. Schmidtke K, Pohlmann S, Metternich B. The syndrome of functional memory disorder: definition, etiology, and natural course. *Am J Geriatr Psychiatry* 2008;**16**:981–8. doi: 10.1097/JGP.0b013e318187ddf9

8. Stone J, Smyth R, Carson A, et al. Systematic review of misdiagnosis of conversion symptoms and "hysteria". *Br Med J* 2005;**331**:989. doi: 10.1136/bmj.38628.466898.55

9. Bailey C, Bell SM, Blackburn DM. How the UK describes functional memory symptoms. *Psychogeriatrics* 2017;**17**: 336–7.

10. Metternich B, Kosch D, Kriston L, et al. The effects of nonpharmacological interventions on subjective memory complaints: a systematic review and meta-analysis. *Psychotherapy and Psychosomatics* 2010;**79**:6–19. doi: 10.1159/000254901

11. Bhome R, Berry AJ, Huntley JD, et al. Interventions for subjective cognitive decline: systematic review and meta-analysis. *BMJ Open* 2018;**8**:e021610. doi: 10.1136/bmjopen-2018-021610

12. Hayes SC, Luoma JB, Bond FW, et al. Acceptance and commitment therapy: Model, processes and outcomes. *Behavior Research and Therapy* 2006;**44**:1–25. doi: 10.1016/j.brat.2005.06.006

For peer review only

13. Cope SR, Poole N, Agrawal N. Treating functional non-epileptic attacks - Should we consider acceptance and commitment therapy? *Epilepsy Behav* 2017;**73**:197-203. doi: 10.1016/j.yebeh.2017.06.003

14. Hayes SC, Luoma JB, Bond FW, Masuda A, Lillis J. Acceptance and commitment therapy: model, processes and outcomes. *Behav Res Ther* 2006;44:1–25. doi: 10.1016/j.brat.2005.06.006.

15. Levin ME, MacLane C, Daflos S, et al. Examining psychological inflexibility as a transdiagnostic process across psychological disorders. *Journal of Contextual Behavioral* Science 2014;**3**:155–63. doi: 10.1016/j.jcbs.2014.06.003

16. Brown RJ, Reuber, M. Towards an integrative theory of psychogenic non-epileptic seizures (PNES). *Clinical Psychology Review* 2016;47:55–70. doi: 10.1016/j.cpr.2016.06.003.

17. Gloster AT, Walder N, Levin ME, et al. The empirical status of acceptance and commitment therapy: A review of meta-analyses. *Journal of Contextual Behavioral Science* 2020;**18**:181-192. doi: 10.1016/j.jcbs.2020.09.009.

18. Du S, Dong J, Jin S, Zhang H, Zhang Y. Acceptance and Commitment Therapy for chronic pain on functioning: A systematic review of randomized controlled trials. *Neurosci Biobehav Rev* 2021;**131**:59-76. doi: 10.1016/j.neubiorev.2021.09.022

19. Graham CD, Gouick J, Krahé C, Gillanders D. A systematic review of the use of Acceptance and Commitment Therapy (ACT) in chronic disease and long-term conditions. *Clinical Psychology Review*, 2016;**46**:46-58. doi: 10.1016/j.cpr.2016.04.009

20. Graham CD, O'Hara DJ, Kemp S. A case series of Acceptance and Commitment Therapy (ACT) for reducing symptom interference in functional neurological disorders. *Clinical Psychology and Psychotherapy*, 2018;25 (3):489-96. doi: 10.1002/cpp.2174

21. Johns LC, Oliver JE, Khondoker M, et al. The feasibility and acceptability of a brief Acceptance and Commitment Therapy (ACT) group intervention for people with psychosis: The 'ACT for life' study. *J Behav Ther Exper Psychiatry* 2016;50:257-63. doi: 10.1016/j.jbtep.2015.10.001

22. Thompson EM, Destree L, Albertella L, Fontenelle LF. Internet-Based Acceptance and Commitment Therapy: A Transdiagnostic Systematic Review and Meta-Analysis for Mental Health Outcomes. *Behavior Therapy* 2021;52(2):492-507. doi: 10.1016/j.beth.2020.07.002

For peer review only

23. World Health Organisation (WHO) (2010). Measuring Health and Disability: Manual for WHO Disability Assessment Schedule (WHODAS 2.0). Edited by TB Üstün, N Kostanjsek, S Chatterji, J Rehm. Geneva (CH): World Health Organization.

24. Herdman M, Gudex C, Lloyd A, Janssen M, Kind P, Parkin D, Bonsel G, Badia X. Development and preliminary testing of the new five-level version of EQ-5D (EQ-5D-5L). Qual Life Res. 2011 Dec;20(10):1727-36. doi: 10.1007/s11136-011-9903-x.

25. Al-Janabi H, Flynn T, Coast J. (2012). Development of a self-report measure of capability wellbeing for adults: the ICECAP-A. Quality of Life Research 21: 167-76. doi: 10.1007/s11136-011-9927-2.

26. Troyer AK, Rich JB (2002). Psychometric properties of a new metamemory questionnaire for older adults. Journal of Gerontology, 57(1), 19-27. doi: 10.1093/geronb/57.1.p19.

27. Kroenke K, Spitzer RL, Williams JB (2001) The PHQ-9: validity of a brief depression severity measure. J Gen Intern Med, 16(9): 606-13. doi: 10.1046/j.1525-1497.2001.016009606.x

28. Spitzer RL, Kroenke K, Williams JBW, Lowe B (2006) A brief measure for assessing generalized anxiety disorder. Arch Inern Med, 166: 1092-7. doi: 10.1001/archinte.166.10.1092.

29. Bond FW, Hayes SC, Baer RA, Carpenter KM, Guenole N, Orcutt HK, Waltz T, Zettle RD. Preliminary psychometric properties of the Acceptance and Action Questionnaire-II: a revised measure of psychological inflexibility and experiential avoidance. Behav Ther. 2011;42(4):676-88. doi: 10.1016/j.beth.2011.03.007.

30. Tyrer P, Cooper S, Salkovskis P, Tyrer H, Crawford M, Byford S, Dupont S, Finnis S, Green J, McLaren E, Murphy D, Reid S, Smith G, Wang D, Warwick H, Petkova H, Barrett B. Clinical and cost-effectiveness of cognitive behaviour therapy for health anxiety in medical patients: a multicentre randomised controlled trial. Lancet. 2014 18;383(9913):219-25. doi: 10.1016/S0140-6736(13)61905-4.

31. Guy W. Clinical Global Impressions (CGI) Scale, Modified. In Rush, John A.; Task Force for the Handbook of Psychiatric Measures (eds.). *Handbook of Psychiatric Measures* (1st ed.). Washington, DC: American Psychiatric Association. 2000. ISBN 978-0-89042-415-5. OCLC 43483679.

32. Buhrman M, Skoglund A, Husell J, Bergström K, Toet al. Guided Internet-Delivered Acceptance and Commitment Therapy for Chronic Pain Patients: A Randomized Controlled

For peer review only

Trial. *Behaviour Research and Therapy* 2013;51(6): 307–15. doi:10.1016/J.BRAT.2013.02.010.

33. Julius SA. Sample size of 12 per group rule of thumb for a pilot study. Pharm Stat, 2005;4:287-91. doi.org/10.1002/pst.185.

34. Lancaster GA, Dodd S, Williamson PR. Design and analysis of pilot studies: recommendations for good practice. J Eval Clin Practice 2004;10:307-12. doi: 10.1111/j..2002.384.doc.x.

35. Sim J, Lewis M. The size of a pilot study for a clinical trial should be calculated in relation to considerations of precision and efficiency. J Clin Epidemiol 2012;65:301-8. doi: 10.1016/j.jclinepi.2011.07.011.

36. Eldridge SM, Chan CL, Campbell MJ, Bond CM, Hopewell S, Thabane L et al. CONSORT 2010 statement: extension to randomised pilot and feasibility trials *BMJ* 2016;355:i5239. doi:10.1136/bmj.i5239

37. Durlak JA. How to select, calculate, and interpret effect sizes. *Journal of Pediatric Psychology* 2009; 34(9): 917-28. doi: 10.1093/jpepsy/jsp004.

38. Braun V, Clarke V. Using thematic analysis in psychology. *Qual Res Psychol* 2006; 3: 77–101.

For peer review only

Figure 1. Study Flow Chart

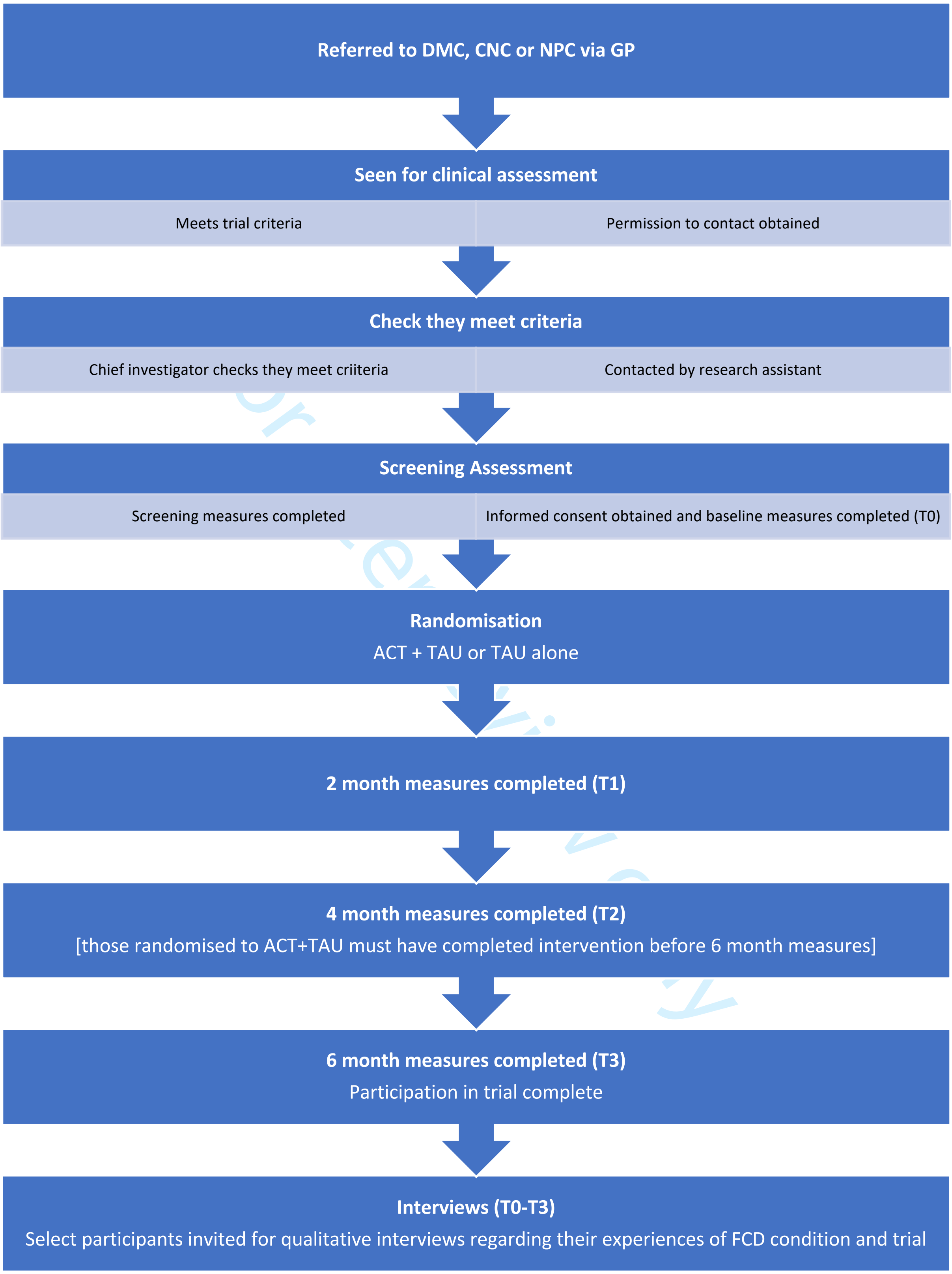

For peer review only - http://bmjopen.bmj.com/site/about/guidelines.xhtml

Reporting checklist for protocol of a clinical trial.

Based on the SPIRIT guidelines.

Instructions to authors

Complete this checklist by entering the page numbers from your manuscript where readers will find each of the items listed below.

Your article may not currently address all the items on the checklist. Please modify your text to include the missing information. If you are certain that an item does not apply, please write "n/a" and provide a short explanation.

Upload your completed checklist as an extra file when you submit to a journal.

In your methods section, say that you used the SPIRITreporting guidelines, and cite them as:

Chan A-W, Tetzlaff JM, Gøtzsche PC, Altman DG, Mann H, Berlin J, Dickersin K, Hróbjartsson A, Schulz KF, Parulekar WR, Krleža-Jerić K, Laupacis A, Moher D. SPIRIT 2013 Explanation and Elaboration: Guidance for protocols of clinical trials. BMJ. 2013;346:e7586

		Reporting Item	Page Number
**Administrative information**			
Title	#1	Descriptive title identifying the study design, population, interventions, and, if applicable, trial acronym	1
Trial registration	#2a	Trial identifier and registry name. If not yet registered, name of intended registry	4
Trial registration: data set	#2b	All items from the World Health Organization Trial Registration Data Set	
Protocol version	#3	Date and version identifier	2
Funding	#4	Sources and types of financial, material, and other support	23

For peer review only

Roles and responsibilities: contributorship	#5a	Names, affiliations, and roles of protocol contributors	22
Roles and responsibilities: sponsor contact information	#5b	Name and contact information for the trial sponsor	22
Roles and responsibilities: sponsor and funder	#5c	Role of study sponsor and funders, if any, in study design; collection, management, analysis, and interpretation of data; writing of the report; and the decision to submit the report for publication, including whether they will have ultimate authority over any of these activities	22
Roles and responsibilities: committees	#5d	Composition, roles, and responsibilities of the coordinating centre, steering committee, endpoint adjudication committee, data management team, and other individuals or groups overseeing the trial, if applicable (see Item 21a for data monitoring committee)	23-24
**Introduction**			
Background and rationale	#6a	Description of research question and justification for undertaking the trial, including summary of relevant studies (published and unpublished) examining benefits and harms for each intervention	5-8
Background and rationale: choice of comparators	#6b	Explanation for choice of comparators	12-14
Objectives	#7	Specific objectives or hypotheses	8
Trial design	#8	Description of trial design including type of trial (eg, parallel group, crossover, factorial, single group), allocation ratio, and framework (eg, superiority, equivalence, non-inferiority, exploratory)	9

For peer review only

Methods: Participants, interventions, and outcomes

Study setting	#9	Description of study settings (eg, community clinic, academic hospital) and list of countries where data will be collected. Reference to where list of study sites can be obtained	9
Eligibility criteria	#10	Inclusion and exclusion criteria for participants. If applicable, eligibility criteria for study centres and individuals who will perform the interventions (eg, surgeons, psychotherapists)	9-10
Interventions: description	#11a	Interventions for each group with sufficient detail to allow replication, including how and when they will be administered	12-14
Interventions: modifications	#11b	Criteria for discontinuing or modifying allocated interventions for a given trial participant (eg, drug dose change in response to harms, participant request, or improving / worsening disease)	n/a
Interventions: adherance	#11c	Strategies to improve adherence to intervention protocols, and any procedures for monitoring adherence (eg, drug tablet return; laboratory tests)	10 & 13
Interventions: concomitant care	#11d	Relevant concomitant care and interventions that are permitted or prohibited during the trial	13-14
Outcomes	#12	Primary, secondary, and other outcomes, including the specific measurement variable (eg, systolic blood pressure), analysis metric (eg, change from baseline, final value, time to event), method of aggregation (eg, median, proportion), and time point for each outcome. Explanation of the clinical relevance of chosen efficacy and harm outcomes is strongly recommended	10-12
Participant timeline	#13	Time schedule of enrolment, interventions (including any run-ins and washouts), assessments, and visits	19-20

For peer review only

		for participants. A schematic diagram is highly recommended (see Figure)	
Sample size	#14	Estimated number of participants needed to achieve study objectives and how it was determined, including clinical and statistical assumptions supporting any sample size calculations	14
Recruitment	#15	Strategies for achieving adequate participant enrolment to reach target sample size	9
**Methods: Assignment of interventions (for controlled trials)**			
Allocation: sequence generation	#16a	Method of generating the allocation sequence (eg, computer-generated random numbers), and list of any factors for stratification. To reduce predictability of a random sequence, details of any planned restriction (eg, blocking) should be provided in a separate document that is unavailable to those who enrol participants or assign interventions	10
Allocation concealment mechanism	#16b	Mechanism of implementing the allocation sequence (eg, central telephone; sequentially numbered, opaque, sealed envelopes), describing any steps to conceal the sequence until interventions are assigned	10
Allocation: implementation	#16c	Who will generate the allocation sequence, who will enrol participants, and who will assign participants to interventions	10 & 19
Blinding (masking)	#17a	Who will be blinded after assignment to interventions (eg, trial participants, care providers, outcome assessors, data analysts), and how	11
Blinding (masking): emergency unblinding	#17b	If blinded, circumstances under which unblinding is permissible, and procedure for revealing a participant's allocated intervention during the trial	11

For peer review only

**Methods: Data collection, management, and analysis**			
Data collection plan	#18a	Plans for assessment and collection of outcome, baseline, and other trial data, including any related processes to promote data quality (eg, duplicate measurements, training of assessors) and a description of study instruments (eg, questionnaires, laboratory tests) along with their reliability and validity, if known. Reference to where data collection forms can be found, if not in the protocol	11
Data collection plan: retention	#18b	Plans to promote participant retention and complete follow-up, including list of any outcome data to be collected for participants who discontinue or deviate from intervention protocols	12
Data management	#19	Plans for data entry, coding, security, and storage, including any related processes to promote data quality (eg, double data entry; range checks for data values). Reference to where details of data management procedures can be found, if not in the protocol	16
Statistics: outcomes	#20a	Statistical methods for analysing primary and secondary outcomes. Reference to where other details of the statistical analysis plan can be found, if not in the protocol	14-16
Statistics: additional analyses	#20b	Methods for any additional analyses (eg, subgroup and adjusted analyses)	14-16
Statistics: analysis population and missing data	#20c	Definition of analysis population relating to protocol non-adherence (eg, as randomised analysis), and any statistical methods to handle missing data (eg, multiple imputation)	14-16
**Methods: Monitoring**			

For peer review only

Data monitoring: formal committee	#21a	Composition of data monitoring committee (DMC); summary of its role and reporting structure; statement of whether it is independent from the sponsor and competing interests; and reference to where further details about its charter can be found, if not in the protocol. Alternatively, an explanation of why a DMC is not needed	18
Data monitoring: interim analysis	#21b	Description of any interim analyses and stopping guidelines, including who will have access to these interim results and make the final decision to terminate the trial	n/a
Harms	#22	Plans for collecting, assessing, reporting, and managing solicited and spontaneously reported adverse events and other unintended effects of trial interventions or trial conduct	17
Auditing	#23	Frequency and procedures for auditing trial conduct, if any, and whether the process will be independent from investigators and the sponsor	n/a
**Ethics and dissemination**			
Research ethics approval	#24	Plans for seeking research ethics committee / institutional review board (REC / IRB) approval	3
Protocol amendments	#25	Plans for communicating important protocol modifications (eg, changes to eligibility criteria, outcomes, analyses) to relevant parties (eg, investigators, REC / IRBs, trial participants, trial registries, journals, regulators)	18
Consent or assent	#26a	Who will obtain informed consent or assent from potential trial participants or authorised surrogates, and how (see Item 32)	9
Consent or assent: ancillary studies	#26b	Additional consent provisions for collection and use of participant data and biological specimens in ancillary studies, if applicable	n/a

For peer review only

Confidentiality	#27	How personal information about potential and enrolled participants will be collected, shared, and maintained in order to protect confidentiality before, during, and after the trial	16
Declaration of interests	#28	Financial and other competing interests for principal investigators for the overall trial and each study site	25
Data access	#29	Statement of who will have access to the final trial dataset, and disclosure of contractual agreements that limit such access for investigators	
Ancillary and post trial care	#30	Provisions, if any, for ancillary and post-trial care, and for compensation to those who suffer harm from trial participation	n/a
Dissemination policy: trial results	#31a	Plans for investigators and sponsor to communicate trial results to participants, healthcare professionals, the public, and other relevant groups (eg, via publication, reporting in results databases, or other data sharing arrangements), including any publication restrictions	18
Dissemination policy: authorship	#31b	Authorship eligibility guidelines and any intended use of professional writers	n/a
Dissemination policy: reproducible research	#31c	Plans, if any, for granting public access to the full protocol, participant-level dataset, and statistical code	22
**Appendices**			
Informed consent materials	#32	Model consent form and other related documentation given to participants and authorised surrogates	Supplement
Biological specimens	#33	Plans for collection, laboratory evaluation, and storage of biological specimens for genetic or molecular analysis in the current trial and for future use in ancillary studies, if applicable	n/a

None The SPIRIT Explanation and Elaboration paper is distributed under the terms of the Creative Commons Attribution License CC-BY-NC. This checklist can be completed online using

For peer review only

https://www.goodreports.org/, a tool made by the EQUATOR Network in collaboration with Penelope.ai

For peer review only

South West London and
St George's Mental Health
NHS Trust

Acceptance and Commitment Therapy (ACT) for Functional Cognitive Disorder (FCD) - ACT4FCD

Chief Investigator: Dr Norman Poole

Participant Information Sheet

We'd like to invite you to take part in our research study. Before you decide, it is important that you understand why the research is being done and what it would involve for you. Please take time to read this information and discuss it with others if you wish. If there is anything that is not clear, or if you would like more information, please contact the research team (see details at the bottom of this leaflet).

What is the aim of this study?

People with Functional Cognitive Disorder (FCD) suffer from memory problems that negatively impact their everyday life and personal wellbeing. At present treatment options are limited in number and accessibility. Acceptance and Commitment Therapy (ACT) focuses on changing a person's relationship with their thoughts and feelings, using mindfulness and acceptance. ACT has been successfully used in the treatment of conditions similar to FCD, and we have tested ACT on FCD in one small study where it appeared to work well.

Now we need to try it with a larger number of patients from different clinics and different backgrounds to make sure it is acceptable and useful to all patients. When we introduce a new way of working it is important that we compare it against our usual way of working and measure the results to see which way is best. To do this we will be allocating half (50%) of all people who take part into the usual care treatment group and half (50%) into the usual care plus specialist ACT group.

To try and make sure the groups are the same to start with, each participant is put into a group by chance (randomly). This process is called randomisation and to ensure that it is fair, the group you will be allocated to will be decided by a computer programme in a clinical trials unit. None of the researchers or members of the care team will have any input into which group you will be allocated to. The findings will help us to further develop specialist 'ACT' support programmes. This is a feasibility study (a practice-run before doing a large-scale study). It will help us find out more about:

For peer review only - http://bmjopen.bmj.com/site/about/guidelines.xhtml

South West London and St George's Mental Health NHS Trust

a. whether people with functional cognitive disorder find this intervention helpful; and how we can improve it.
b. whether people who have functional cognitive disorder find this type of trial acceptable and whether they are willing to be randomly allocated to receive the specialist support we have developed, in addition to their usual care; or receive only the usual care that is currently available to them.
c. how best to train staff to deliver this specialist support.
d. how best to measure the costs of that support and how well it works.
e. whether people taking part can complete the questionnaires we plan to use, without difficulty

Once we have completed the study and gathered information about each of these points, we can then make changes to prepare for conducting a larger study. We need to ensure that research meets patient needs and are asking your help to do this. If you are interested in taking part, please read the rest of this information sheet.

Why have I been invited?
We are asking you to take part because you have an established diagnosis of functional cognitive disorder that resulted in using one of the outpatient clinics and you are aged 18 or over. Your consultant has helped us to identify who to ask. We are inviting a total of 48 participants like you to take part.

Do I have to take part?
No. It is up to you to decide whether or not to take part. If you do decide to take part you will be
given this information sheet to keep and be asked to sign a consent form. If you decide to take part you are still free to withdraw at any time and without giving a reason. This would not affect your legal rights.

What will happen to me if I take part?
After you have consented, the research assistant will conduct an initial assessment to confirm you are eligible to take part in the study. If found to be eligible, you will be asked to complete several questionnaires assessing your quality of life, mood, memory, and how well you are functioning with the condition.

After this initial assessment the trial manager will contact King's College clinical trials unit (an external provider) who will allocate (randomise) you into one of the two groups, either the ACT intervention or standard medical care. The trial manager will then contact you to let you know which group you have been allocated to.

For peer review only - http://bmjopen.bmj.com/site/about/guidelines.xhtml

South West London and St George's Mental Health NHS Trust

Whichever arm of the trial you are allocated to, you will be asked to complete questionnaires on 4 separate occasions; at baseline, and again 2 months, 4 months and finally at 6 months after first being randomised. After completing them on this 4th and final occasion you will leave the study.

The questionnaires allow us to study whether the ACT intervention has been helpful and whether any improvements are maintained beyond the end of the intervention. A member of the research team will be on hand to assist with completing the questionnaires, should this be required. Each set of questionnaires will take between 35 - 45 minutes to complete.

What are the interventions I could receive?

You will receive either:

a. the usual care and access to services provided for people following a diagnosis of functional cognitive disorder within this Trust (consisting of the standard care already provided to you in the local memory clinics, neuropsychiatry service, or cognitive neurology clinic (wherever you were seen for assessment and diagnosis).
b. the usual care and access to services provided for people following functional cognitive disorder within this Trust **plus** the specialist ACT intervention being tested in this study.

There is a possibility that you may be disappointed by which group you have been allocated to, but each of the groups **is equally important** to developing this specialist ACT support programme and we hope that whatever the outcome you will continue to take part.

<u>During the 6 months in the study</u>

If you are allocated to the usual care group you will receive all the usual support and access to services provided by your care team.

If you are allocated to receive the specialist ACT programme from the clinician, you will be contacted by the treating clinician to arrange for the group intervention which consists of 4, 2-hour weekly treatment sessions, followed by 1, 2-hour booster session a month after the final treatment session. The ACT Group Intervention will host roughly 5-8 participants per group and will be entirely online. You will be expected to attend all 5 sessions if allocated to this group. Over the study period we will run groups on different days of the week and times of the day to maximise the chance of there being a group convenient to you. However, once

For peer review only - http://bmjopen.bmj.com/site/about/guidelines.xhtml

South West London and St George's Mental Health NHS Trust

allocated a particular group you will remain in that one for the duration of your intervention. They are run online to minimise disruption to your schedule.

The ACT intervention includes education about normal memory and forgetting. The intervention aims to decrease the threat of memory failures and to alter a person's relationship to their symptoms while encouraging behaviour that is in line with their values and goals. The concepts of "primary suffering" (the unwanted memory symptoms) and "secondary suffering" (attempts to control memory failures) are discussed. Brief mindfulness practices are incorporated into the sessions to improve acceptance, aiming for less distressing interpretations of unwanted experiences. Value-based goals are identified throughout the sessions to shift away from avoidance-based behaviour and the focus on memory symptoms.

What happens during a session?

All the sessions are run by two of the researchers who have expertise in delivering online group psychological therapies. In each of the sessions, specific topics are covered, and exercises undertaken. Each session begins with an overview of what will be covered and a reminder of what had been discussed at the previous one. Members of the group are encouraged to participate openly in these sessions and share their thoughts and experiences. Homework is set at the end of each session and discussed at the subsequent one. At the end of each session some tasks are given for trying out before the next session. Feedback is then shared at the next session. Participants are actively encouraged to engage in sessions as this appears to strengthen positive outcomes of the treatment. Everything discussed is confidential so must not be shared outside the group, and this will be explained again at the start of each session.

Recording of Sessions

All sessions of the ACT Intervention will be video recorded using MS Teams. The purpose of recording is not to review participants, but to ensure that the ACT process has been followed. The recordings will be reviewed for ACT fidelity and deleted at the end of the trial. Participants have the right to switch off their camera during sessions if they do not wish to be recorded.

Follow-up

While in the study (regardless of which group you are in) we would like to follow your progress and will ask you to complete questionnaires (either online or paper copies can be sent to you) at the **start** of the study and again at **2**, **4** and **6** months. When these questionnaires are due the research assistant will contact you to confirm that you are happy to continue and arrange a mutually convenient time to meet with you in person or online to support you in completing

For peer review only - http://bmjopen.bmj.com/site/about/guidelines.xhtml

South West London and St George's Mental Health NHS Trust

the questionnaires. These questionnaires assess how you are doing so are very important for the study.

We will also collect information from you and your medical notes about the amount of support you have received previously and which services you have accessed. We will only collect information directly relevant to your participation in this study and nothing else.

Additionally, as part of a sub-study, we may wish to interview you for about 45-60 minutes face-to-face, either via an online videocall or in person. This interview will be audio recorded and then transcribed. Like all of your data, the recording and transcription will be anonymised. We will ask you about the support you have received and the things you found useful or most helped you in your day-to-day functions. If you are invited to attend the interview, this will be a one-off occurrence and entirely voluntary, and you will be given further information beforehand so that you can decide whether you would like to be interviewed or not. **You do not have to agree to this interview to be able to take part in the study.**

Travel expenses and payment for participation

Participants will receive £25 for taking part in the trial, plus reimbursement of any travel costs involved, and £10 for completing each full set of questionnaires. We will also pay £20 to those who take part in the additional 45-60 minutes interview.

What are the possible disadvantages and risks of taking part?

We do not expect there are any disadvantages or risks to you. We will arrange any interviews at times to suit you. You may feel anxious before or tired after taking part in the sessions or while completing the questionnaires, but we will do everything we can to minimise or prevent this. You will be asked about your well-being over the course of the study, and you will have the opportunity to take short rest breaks when completing the questionnaires.

You may find the ACT sessions tiring or inconvenient, however, the timing of these will be arranged to be maximally convenient to participants. We will run groups on two days of the week at different times so if one group runs at a time that is inconvenient then you will have the option of an alternative day/time.

What are the possible benefits of taking part?

We cannot promise that being in either arm of this trial will help you but the information we obtain will help us plan a larger study to test how effective the ACT intervention is. In the future, this could help improve services for other people who have a functional cognitive disorder.

For peer review only - http://bmjopen.bmj.com/site/about/guidelines.xhtml

South West London and St George's Mental Health NHS Trust

What happens when the study stops?

When the study ends after 6 months, you will continue with your usual care from your hospital or GP.

What will happen if I don't want to carry on with the study?

Your participation is voluntary, and you are free to withdraw at any time, without giving any reason, and without your legal rights being affected. If you withdraw then the information collected so far cannot be erased and this information may still be used in the project analysis.

What if there is a problem?

If you have a concern about any aspect of this study, you should ask to speak to the Principal Investigator Dr Norman Poole or Trial Manager Dr Serena Vanzan (contact details below) who will do their best to answer your questions.

If you remain unhappy and wish to complain formally, you can do this through the NHS Complaints Procedure. Details can be obtained from the Trust's Patient Advice and Liaison Service (PALS). Tel: 0203 513 6150 (Monday - Friday 9.30am to 4.30pm) or email pals@swlstg.nhs.uk.

How will we use information about you?

We will need to use information from your medical records for this research project. This information will include your:

- NHS/Hospital number
- Name
- Contact details
- Ethnicity
- Gender
- DoB
- Relationship status
- Employment status
- Current medication
- Medical history

People will use this information to do the research or to check your records to make sure that the research is being done correctly. People who do not need to know who you are will not be able to see your name or contact details. Your data will have a code number instead. We will keep all information about you safe and secure.

Once we have finished the study, we will keep some of the data so we can check the results. We will write our reports in a way that no-one can work out that you took part in the study.

For peer review only - http://bmjopen.bmj.com/site/about/guidelines.xhtml

NHS
South West London and
St George's Mental Health
NHS Trust

What are your choices about how your information is used?

You can stop being part of the study at any time, without giving a reason, but we will keep information about you that we already have.

- We need to manage your records in specific ways for the research to be reliable. This means that we won't be able to let you see or change the data we hold about you.
- If you agree to take part in this study, you will have the option to take part in future research using your data saved from this study. If you consent to this we may:
    - Use your data already collected for this study in future research.
    - Contact you regarding taking part in future research relating to this current study.

Where can you find out more about how your information is used?

You can find out more about how we use your information

- at www.hra.nhs.uk/information-about-patients/
- by asking one of the research team
- by sending an email to SWLSTG's Data Protection Officer, John Hughes (john.hughes@swlstg.nhs.uk)
- by ringing us on 020 8725 3786

Will my taking part in this study be kept confidential?

We will follow ethical and legal practice and all information about you will be handled in confidence.

If you join the study, some parts of your medical records and the data collected for the study will be looked at by authorised persons from South West London Mental Health Trust, who are organising the research, and the King's Clinical Trials Unit. It may also be looked at by authorised people to check that the study is being carried out correctly. All will have a duty of confidentiality to you as a research participant and we will do our best to meet this duty.

All information which is collected about you during the course of the research will be kept strictly confidential, stored in a secure and locked office, and on a password protected database. Any information about you which leaves the hospital will have your name and address removed (anonymised) and a unique code will be used so that you cannot be recognised from it.

Your personal data (address, telephone number) will be kept for 6 months after the end of the study so that we are able to contact you about the findings of the study and possible follow-up studies (unless you advise us that you do not wish to be contacted). All other data

For peer review only - http://bmjopen.bmj.com/site/about/guidelines.xhtml

South West London and St George's Mental Health NHS Trust

(research data) will likewise be kept securely for 5 years after completion of the whole study (not just your involvement in it). After this time your data will be disposed of securely. During this time all precautions will be taken by all those involved to maintain your confidentiality, only members of the research team will have access to your personal data.

Although the information we collect about you is confidential, should you disclose anything to us which we feel puts you or anyone else at risk, we may feel it necessary to report this to the appropriate persons.
We would ask for your permission for the anonymised data set to be used to inform future projects and for education purposes and this permission is included within your consent form.

Involvement of the General Practitioner (GP)
If you do decide to take part in the study, we will inform your GP and provide them with a copy of this information sheet.

What will happen to the results of the research study?
We will use these findings to support the design of a large-scale study to test whether this ACT intervention results in people with functional cognitive disorder having better quality lives. The findings will be written up and submitted for publication to enable other NHS services to learn from our experiences. All reports and publications will be anonymised, and you would not be identified in any report or publication.

Who is organising and funding the research?
The study is being organised by the South West London and St Georges Mental Health NHS Foundation Trust (SWLSTG) and is being funded by National Institute for Health and Care Research (NIHR) Programme (project number NIHR202743).

Who has reviewed the study?
All research in the NHS is looked at by independent groups of people, called a Research Ethics Committee to protect your interests. This study has been reviewed and received a favourable ethical opinion from South East Scotland REC 02 (22-SS-0059).

Further information and contact details
If you have any questions about the study, wish to discuss taking part or have any concerns, you can contact the researchers leading the study:

Chief Investigator	Dr Norman Poole	act4fcd@swlstg.nhs.uk	07519668140
Trial Manager	Dr Serena Vanzan		
Research Assistant	Aimee Duffus		

For peer review only - http://bmjopen.bmj.com/site/about/guidelines.xhtml

South West London and St George's Mental Health NHS Trust

General information about taking part in research studies can also be obtained from your hospital's Patient Advice and Liaison Service (PALS), Tel: 0203 513 6150 (Monday - Friday 9.30am to 4.30pm) or email pals@swlstg.nhs.uk.

Many thanks for reading this information sheet. Please keep this information sheet. We will ask you to sign a consent form if you agree to take part and we will give you a copy of it to keep.

For peer review only

For peer review only - http://bmjopen.bmj.com/site/about/guidelines.xhtml

South West London and St George's Mental Health NHS Trust

Acceptance and Commitment Therapy (ACT) for Functional Cognitive Disorder (FCD)- ACT4FCD

Chief Investigator: Dr Norman Poole

Informed Consent Form

Participant Number:____________________

Please initial box

1. I confirm that I have read the information sheet dated 16.11.22 (version 2.1) for this study. I have had the opportunity to consider the information, ask questions and have had these answered satisfactorily. ☐

2. I understand that my participation is voluntary and that I am free to withdraw at any time without giving any reason, without my medical care or legal rights being affected. ☐

3. I understand that the data collected up to the point of my withdrawal will still be used for analysis. ☐

4. If randomly allocated to the ACT intervention group, I consent to be video recorded during therapy session. ☐

5. I understand that relevant sections of my medical notes and data collected during the study, may be looked at by individuals from the research team, where it is relevant to my taking part in this research. I give permission for these individuals to have access to my records. ☐

6. I understand my personal data will be kept for a maximum of 6 months following the end of the study and anonymised data will be made available to the research team to support future research. ☐

7. I agree to my General Practitioner being informed of my participation in the study. ☐

For peer review only

For peer review only - http://bmjopen.bmj.com/site/about/guidelines.xhtml

South West London and St George's Mental Health NHS Trust

8. I agree to take part in this study. ☐

9. I agree to be contacted to take part in an additional interview with the research team to share my experiences of living with FCD and of taking part in this trial. I understand that agreeing to be contacted does not oblige me to participate in the additional interview. Yes ☐ No ☐

10. I agree to be contacted to take part in future research regarding treatment for FCD. I understand that agreeing to be contacted does not oblige me to participate in any further studies. Yes ☐ No ☐

11. I would like to receive the study outcomes at the end of the study. Yes ☐ No ☐

**Name of Participant**	**Date**	**Signature**
Name of Person taking consent	Date	Signature

For peer review only - http://bmjopen.bmj.com/site/about/guidelines.xhtml
For peer review only

Summary of ACT for FCD Group Intervention

The group includes psychoeducation on normal memory functioning, in particular the role of attention and normal patterns of forgetting. This psychoeducation element aims to reduce the threat of cognitive symptoms. In line with the ACT model, the group aim is to increase psychological flexibility in response to the symptoms and associated thoughts and feelings. ACT is a contextual cognitive-behavioural therapy approach. Instead of targeting specific thoughts in relation to unwanted symptoms, it focuses on altering a person's relationship with their symptoms, increasing acceptance of the symptoms, and encouraging behaviour that is in line with a person's values (moving away from behaviour that focuses on avoidance of symptoms).

The concepts of "primary suffering" (the unwanted cognitive symptoms) and "secondary suffering" (attempts to control cognitive "failures" causes additional suffering, such as ruminations, negative predictions, and avoidance) are introduced. It is explicitly stated that the intervention does not aim to reduce "primary suffering" (cognitive symptoms), although it is possible that improvements may occur if secondary suffering is reduced. Brief mindfulness practices are incorporated in the group to facilitate "bottom up" processing, acceptance, and more neutral interpretations of unwanted experiences. Value-based goals are identified throughout the sessions to shift away from avoidance-based behaviour and the focus on cognitive symptoms.

The intervention protocol will be amended in light of specific feedback received during the contemporaneous qualitative interviews which are designed to explore the participants experience of and satisfaction with the groups.

Summary of the session content

	Group content
**Session 1**	Ground rules/house-keeping<br>Introductions<br>Psychoeducation (models of memory, normal forgetting, attention, functional cognitive disorder, fight/flight response)<br>Primary and secondary suffering<br>Vicious cycles in FCD<br>Brief mindfulness practice<br><br>Home practice:<br>Read Session 1 handout and write down any questions
**Session 2**	Brief mindfulness practice

For peer review only

	Check-in regarding home practice<br>What is ACT<br>Mindfulness<br>Values<br><br>Home-practice:<br>Mindfulness practice<br>Read Session 2 handout and write down any questions
**Session 3**	Brief mindfulness practice<br>Check-in regarding home practice<br>Value-based action<br>Value-based goals (passengers on the bus metaphor)<br><br>Home practice:<br>Working towards value-based goal<br>Mindfulness practice<br>Read Session 3 handout and write down any questions
**Session 4**	Brief mindfulness practice<br>Check-in regarding home practice<br>Cognitive fusion: Thoughts as barriers<br>Complete Willingness & Action plan<br><br>Home practice:<br>Working towards value-based goal<br>Mindfulness practice<br>Read Session 4 handout and write down any questions
**Session 5 (booster)**	Review goals<br>Trouble-shooting

Summary of ACT for FCD Group Intervention

The group includes psychoeducation on normal memory functioning, in particular the role of attention and normal patterns of forgetting. This psychoeducation element aims to reduce the threat of cognitive symptoms. In line with the ACT model, the group aim is to increase psychological flexibility in response to the symptoms and associated thoughts and feelings. ACT is a contextual cognitive-behavioural therapy approach. Instead of targeting specific thoughts in relation to unwanted symptoms, it focuses on altering a person's relationship with their symptoms, increasing acceptance of the symptoms, and encouraging behaviour that is in line with a person's values (moving away from behaviour that focuses on avoidance of symptoms).

The concepts of "primary suffering" (the unwanted cognitive symptoms) and "secondary suffering" (attempts to control cognitive "failures" causes additional suffering, such as ruminations, negative predictions, and avoidance) are introduced.

It is explicitly stated that the intervention does not aim to reduce “primary suffering” (cognitive symptoms), although it is possible that improvements may occur if secondary suffering is reduced. Brief mindfulness practices are incorporated in the group to facilitate “bottom up” processing, acceptance, and more neutral interpretations of unwanted experiences. Value-based goals are identified throughout the sessions to shift away from avoidance-based behaviour and the focus on cognitive symptoms.

The intervention protocol will be amended in light of specific feedback received during the contemporaneous qualitative interviews which are designed to explore the participants experience of and satisfaction with the groups.

For peer review only

NHS
South West London and St George's Mental Health NHS Trust

Data Management Plan (DMP)

Acceptance and Commitment Therapy (ACT) for Functional Cognitive Disorder (FCD) - ACT4FCD

POON1002

1. Trial information

Trial type	Non-CTIPM Intervention - Feasibility
Study Sites	Single-site (SWLSTG)
Total sample size	48
Total duration of study<br><br>(months)	Trial start date _01/05/2022__<br><br>Planned duration for recruitment (months)___6____<br><br>Planned duration of follow-up (months)___6____<br><br>Total Duration ___24____Months
Study objective and design	We aim to study the feasibility of delivering a Randomised Control Trial (RCT) of an online group intervention based on Acceptance and Commitment Therapy (ACT) adapted for those with FCD. The treatment aims to reduce the threat of the memory failures and improve quality of life despite their presence. Participants will be recruited via SWLSTG and St George's neuropsychiatry services and memory clinics. All those eligible will be randomly allocated to either 5 sessions of online group ACT for FCD or treatment as usual. Questionnaires measuring various dimensions of physical, cognitive and mental wellbeing will be administered 2, 4 and 6 months after initial baseline. We will compare the outcome of those who receive the intervention with treatment as usual.

For peer review only - http://bmjopen.bmj.com/site/about/guidelines.xhtml

South West London and St George's Mental Health NHS Trust

	A selection of participants will be invited to take part in interviews about their lived experiences of FCD as well as to receive feedback on the study intervention.
Primary outcome measure	Increased psychological flexibility
Secondary outcome measures	Self-evaluations (questionnaires) of:<br>• Memory<br>• Anxiety<br>• Depression<br>• Global functioning<br>• Quality of life<br>• General wellbeing<br>• Service utilisation
Interim analysis	Yes/**no**, if yes when_____________(Months from baseline)

2. Trial personnel and contact details

This section details the name, their position in the trial, email address, telephone/fax number for all staff involved in the trial including the sponsor. The trial coordinator/trial manager, the investigators, study staff involved in the data management (including computing staff responsibilities for maintaining hardware and software), the monitors and anyone else associated with the trial at each site.

2.1 Sponsor site personnel (add or remove accordingly)

Role	Name	Organisation	Contact Details
Sponsor	SWLSTG R&D	SWLSTG	ResearchDevelopment@swlstg.nhs.uk
Chief Investigator	Norman Poole	SWLSTG	Norman.Poole@swlstg.nhs.uk
Trial Manager	Serena Vanzan	SWLSTG	Serena.Vanzan@swlstg.nhs.uk
Research Assistant	Aimee Duffus	SWLSTG	Aimee.Duffus@swlstg.nhs.uk
Research Assistant	Rebecca Cox	SWLSTG	Rebecca.Cox@swlstg.nhs.uk
Research Assistant	Tasnim Fakira	SWLSTG	Tasnim.Fakira@swlstg.nhs.uk
Co-Investigator and Trial statistician	Jared Smith	SGUL	jasmith@sgul.ac.uk
Co-Investigator and Trial statistician	Nadia Mantovani	SGUL	nmantova@sgul.ac.uk
Co-Investigator	Sarah Cope	SWLSTG	Sarah.Cope@swlstg.nhs.uk

For peer review only - http://bmjopen.bmj.com/site/about/guidelines.xhtml

South West London and St George's Mental Health NHS Trust

Co-Investigator	Mark Edwards	KCL	mark.j.edwards@kcl.ac.uk
Trial statistician	Barbara Barrett	KCL	barbara.m.barrett@kcl.ac.uk
Primary contact for DM issues	Serena Vanzan	SWLSTG	Serena.Vanzan@swlstg.nhs.uk
Secondary contact for DM issues	Norman Poole	SWLSTG	Norman.Poole@swlstg.nhs.uk

For peer review only

For peer review only - http://bmjopen.bmj.com/site/about/guidelines.xhtml

South West London and St George's Mental Health NHS Trust

3. Milestones

3.1 Study Milestones

Milestone	Date/Estimated Date
Date funding confirmed	23.07.2021
Date and version number of approved protocol	14.07.2022, v1.0
Date and version number of final protocol amendment(s)	n/a
Date/version number of final approved CRF	No approval required
Release date/version number of final database	Est end Oct 2022
Date DMP signed off	
Date of first participant first visit (FPFV)	Est 17.10.2022
Date last participant last visit (LPLV)	Est Sep 2023

3.2 Proposed Data Milestones

Milestone	Date/Estimated Date
Data entry to commence	n/a (data collected directly on database)
Date of interim data partial lock if applicable	n/a
Data entry completed	Est Sept 2023
Last query resolved in the system	Est Oct 2023
Date of database final lock	Est Oct 2023

4. Data collection & data entry system

4.1 *Detail how data will be collected and entered from each site, whether to* ***complete the paper CRFs*** *or how to enter data electronically from each site.*

CRFs and outcome measures (questionnaires) will be completed electronically via the software REDCap, either by the RA (CRFs) or the participant (questionnaires). Where the participant will prefer paper questionnaires, these will be provided and returned via post, and the RA will enter the responses onto REDCap.

In the event that the online database will not be finalised at the time of recruitment opening, paper CRF and Questionnaires will be completed by the RA and participants, and the data will be entered onto REDCap as soon as this will become available.

Interview data (audio) will be automatically transcribed into a Word file by Teams, and the files will be saved in the study folder on SWLSTG's shared drive.

4.2 *Provide details on the system used for data entry*

The REDCap online software will be used for collecting CRF and quantitative data.

4.3 *Outline who will be conducting the data entry from each site?*

The RAs and the participants themselves.

For peer review only - http://bmjopen.bmj.com/site/about/guidelines.xhtml

4.4 *Outline whether single or double data entry will be carried out for all sites*
Single.

4.5 *If double data entry is required describe the process and whether a 100% of CRFs or a sample of CRFs will be double entered. Outline how the two entries will be compared, who will carry out the comparison and how the results will be dealt with.*
N/a

4.6 *Outline the checks undertaken for outcome measures*
The TM will perform regular self-monitoring visits in which the data entered for a proportion of participants will be checked for completeness, accuracy and timely completion.

4.7 *Provide details on how the data will be centralised*
N/a – one site.

5. Data checks & data validation for each site

5.1 *Outline who will perform data cleaning, missing data checks, consistency checks, range checks and logic, and how these are checked at each site?*
The TM will perform regular self-monitoring visits in which the data entered for a proportion of participants will be checked for completeness, accuracy and timely completion. In addition, value range restrictions will be applied in REDCap so that no values outside the available range can be entered when completing closed questions.

5.2 *Describe how regularly the data will be checked*
Monthly.

5.3 *Provide contact person for each site for data queries if data managed centrally*
N/a

5.4 *Describe the data flow from each site to central data centre, and who will conduct the overall data check*
N/a – one site.

5.5 *Detail the* ***flow of the data*** *from the field to the final storage for each site*
Quantitative data will be collected directly on study database. Qualitative data (interviews) will be recorded on Teams (on a Trust laptop), transcribed by Teams and saved on the study's shared drive folder.

6. AE and SAE data handling

Outline how the AE data will be collected by each site and collated for all sites at the end of the trial.
N/a – one site.

7. Partial and/or final data check and database lock

For peer review only - http://bmjopen.bmj.com/site/about/guidelines.xhtml

Detail if an interim analysis is planned in the trial protocol, stating the time point and whether the database will be partially locked for the interim analysis.
N/a

Detail the process for partial and final data checks and data lock, outline who checks the data and who will sign off for partial/final data lock form(s)?
The database will be locked after the following actions have been confirmed as completed by the TM:

1. All CRF data has been collected and entered onto REDCap.
2. All queries identified during regular data checks and self-monitoring visits have been resolved/clarified.
3. All missing information has been confirmed as being not available (as opposed to not entered).
4. All monitoring visits (incl. close-out) have been performed, and outstanding actions completed.
5. A final data quality check has been performed.

The TM will be responsible for locking the database (notifying the CI and Sponsor), send the relevant data to the trial statisticians (with treatment allocation coded to prevent unblinding, where relevant), and save a copy of the full database in the TMF.

8. Data security and transmission between sites

Provide details on the data security procedures for transmitting data between sites
To comply with SWLSTG's Information Governance regulations, the datasets will be sent via encrypted email to the trial statisticians.

9. Data export & analysis

Explain how data will be exported and who the data should be sent to for data analysis.
The qualitative data will be saved on Word files, sent via an encrypted email to Nadia Mantovani, Trial Statistician and Co-Investigator. The full database with demographic and questionnaire data will be exported in CSV format and sent via encrypted email to Jared Smith, Trial Statistician and Co-Investigator. The responses to the AD-SUS questionnaire will be exported in Excel format and sent via encrypted email to Barbara Barrett, Trial Statistician.

10. Data Back-up and archiving

Describe procedures in place to ensure data protection including back-up system (if you don't do this you could lose the data!)
The database (all data on REDCap) will be downloaded and backed up weekly on a Trust external hard drive. The TMF, including the interview transcriptions, will be saved on the Trust's shared drive, which is backed up every night, in a restricted folder dedicated to the study. In addition, the TMF will also be backed up once a week on the Trust encrypted external hard drive.

For peer review only - http://bmjopen.bmj.com/site/about/guidelines.xhtml

For peer review only
