# Supplementary material for: Feasibility randomised controlled trial of online group Acceptance and Commitment Therapy for Functional Cognitive Disorder (ACT4FCD)
Source: BMJ Open. 2023 May 11;13(5):e072366. doi: 10.1136/bmjopen-2023-072366 (PMC10186420; doi:10.1136/bmjopen-2023-072366)
Supplement: Reviewer comments [file bmjopen-2023-072366.reviewer_comments.pdf]

PEER REVIEW HISTORY

BMJ Open publishes all reviews undertaken for accepted manuscripts. Reviewers are asked to complete a checklist review form (**http://bmjopen.bmj.com/site/about/resources/checklist.pdf**) and are provided with free text boxes to elaborate on their assessment. These free text comments are reproduced below.

ARTICLE DETAILS

**TITLE (PROVISIONAL)**	Feasibility randomised controlled trial of online group Acceptance and Commitment Therapy for Functional Cognitive Disorder (ACT4FCD)
**AUTHORS**	Poole, Norman; Cope, Sarah; Vanzan, Serena; Duffus, Aimee; Mantovani, Nadia; Smith, Jared; Barrett, Barbara; Tokley, Melanie; Scicluna, Martin; Beardmore, Sarah; Turner, Kati; Edwards, Mark; Howard, Robert

This article was not externally reviewed at BMJ Open. Protocols that have been independently assessed prior to submission to BMJ Open are usually fast-tracked to publication on the grounds that further substantial changes will not be possible. This independent assessment will usually be external, independent review for both a substantial grant award from a non-commercial or government funder and ethics approval.
